# Simultaneous electroencephalography-functional magnetic resonance imaging for assessment of human brain function

**DOI:** 10.3389/fnsys.2022.934266

**Published:** 2022-07-28

**Authors:** Elias Ebrahimzadeh, Saber Saharkhiz, Lila Rajabion, Homayoun Baghaei Oskouei, Masoud Seraji, Farahnaz Fayaz, Sarah Saliminia, Seyyed Mostafa Sadjadi, Hamid Soltanian-Zadeh

**Affiliations:** ^1^School of Electrical and Computer Engineering, College of Engineering, University of Tehran, Tehran, Iran; ^2^School of Cognitive Sciences, Institute for Research in Fundamental Sciences (IPM), Tehran, Iran; ^3^Department of Pharmacology-Physiology, Faculty of Medicine, University of Sherbrooke, Sherbrooke, Canada; ^4^School of Graduate Studies, State University of New York Empire State College, Manhattan, NY, United States; ^5^School of Medicine, Tehran University of Medical Sciences, Tehran, Iran; ^6^Department of Psychology, University of Texas at Austin, Austin, TX, United States; ^7^Department of Biomedical Engineering, School of Electrical Engineering, Payame Noor University of North Tehran, Tehran, Iran

**Keywords:** simultaneous EEG-fMRI, functional neurological disorders, functional connectivity, cognitive neural networks, multimodal image analysis

## Abstract

Electroencephalography (EEG) and functional Magnetic Resonance Imaging (MRI) have long been used as tools to examine brain activity. Since both methods are very sensitive to changes of synaptic activity, simultaneous recording of EEG and fMRI can provide both high temporal and spatial resolution. Therefore, the two modalities are now integrated into a hybrid tool, EEG-fMRI, which encapsulates the useful properties of the two. Among other benefits, EEG-fMRI can contribute to a better understanding of brain connectivity and networks. This review lays its focus on the methodologies applied in performing EEG-fMRI studies, namely techniques used for the recording of EEG inside the scanner, artifact removal, and statistical analysis of the fMRI signal. We will investigate simultaneous resting-state and task-based EEG-fMRI studies and discuss their clinical and technological perspectives. Moreover, it is established that the brain regions affected by a task-based neural activity might not be limited to the regions in which they have been initiated. Advanced methods can help reveal the regions responsible for or affected by a developed neural network. Therefore, we have also looked into studies related to characterization of structure and dynamics of brain networks. The reviewed literature suggests that EEG-fMRI can provide valuable complementary information about brain neural networks and functions.

## Introduction

Understanding the neural basis of brain functioning requires knowledge about the temporal and spatial aspects of information processing. Electrophysiology, and specifically electroencephalography (EEG), constitutes to be a major tool for studying neural basis of brain functioning. Contrary to imaging techniques, EEG provides high temporal resolution and records the electrical activity of the brain in the order of milliseconds, using electrodes positioned on the scalp (Amoozegar et al., 2013; Ebrahimzadeh et al., 2013, 2018b; Mirbagheri et al., 2019). EEG signal processing methods are applied to determine the quantitative parameters on the spectrum of frequencies, amplitudes, and coherence.

Computed tomography (CT), and mainly MRI (Nikravan et al., 2016; Ebrahimzadeh et al., 2018a), on the other hand, represents a morphological view of brain with a significant spatial resolution. It provides a multiparametric evaluation of the brain tissue with respect to both its structural and functional properties. In this context, similar to EEG but at different temporal scales (milliseconds vs. seconds), functional Near Infrared Spectroscopy (fNIRS) (Mirbagheri et al., 2019, 2020a,2020b) measures the hemodynamic response, that is the change of oxygen in the blood when a brain region becomes active and functional MRI (fMRI) (Ebrahimzadeh et al., 2019, 2019a; Sadjadi et al., 2022) offers the possibility for examining the brain functional activation non-invasively both during resting state and task execution, expanding the panel of parameters obtainable by MRI [e.g., structural connectivity evaluated by diffusion tensor imaging (DTI), metabolites concentrations evaluated by magnetic resonance spectroscopy (MRS), and perfusion valuated by arterial spin labeling (ASL)] (Mele et al., 2019). This complementarity of information is highlighted in multimodal recording methods that are designed to overcome the single modality shortcomings and to enhance the patient’s treatment experience. Both in preclinical and clinical settings (Grassi et al., 2009; Greco et al., 2013; Auletta et al., 2017; Aiello et al., 2018; Russo et al., 2018), first multimodal imaging techniques attempted to combine functional information obtained from nuclear medicine modalities (positron emission tomography—PET, and single photon emission computed tomography—SPECT) with structural data achieved by CT and MRI, were performed in order to complement diagnostic and prognostic procedures (Bruno et al., 2011). In neurology, simultaneous PET/MRI paved the way for a more comprehensive investigation of the organization and physiology of the brain and to explore, within a single integrated exam, the cerebral connectivity in terms of structural, functional, and metabolic connectome (Salmon et al., 2015; Aiello et al., 2016).

Recently, novel tools have been designed to acquire fMRI and EEG at the same time in attempts to investigate the brain function thoroughly (Mele et al., 2019). Recording these two signals simultaneously combines the optimal spatial and temporal data of both modalities which overcomes the restrictions of single modalities (Ebrahimzadeh et al., 2019b,2021a,2021b). This approach shows promising potential in providing a more thorough understanding of the underlying mechanisms of neural activities, addressing brain function and dysfunction (Mele et al., 2019).

This paper aims to review the existing literature on the integration of fMRI and EEG. We briefly discuss the complementary features of the two modalities, then move on to methods of acquiring them at the same time. We also describe the positive and negative sides of EEG-fMRI simultaneous acquisition and highlight the strategies employed to ensure their optimal combination.

**FIGURE 1:**
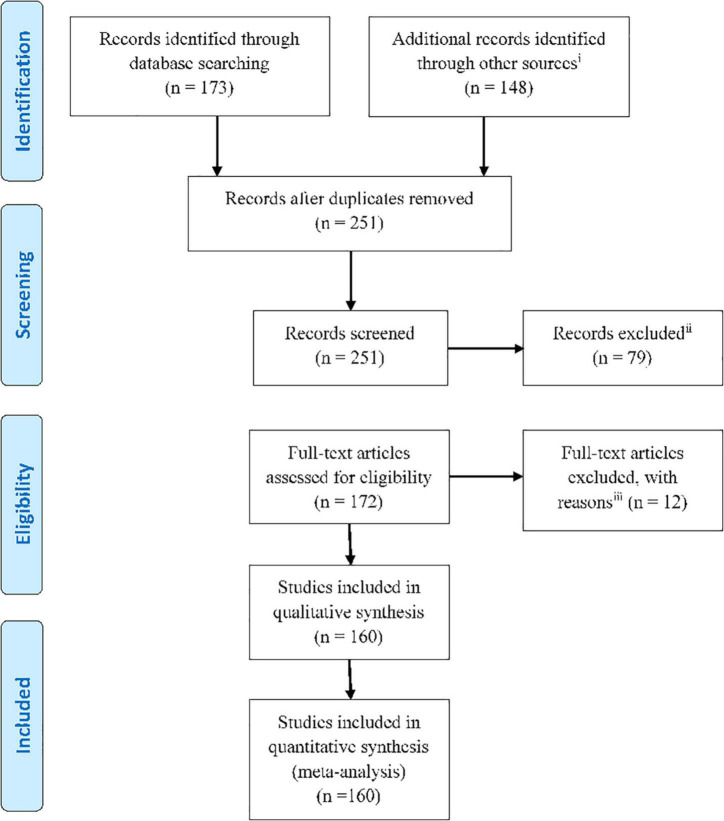
PRISMA flowchart showing the classification of the extracted articles related to the functional neurological assessment by simultaneous EEG-fMRI recording.

We have reviewed a large strand of literature which focuses on functional neurological assessment. The articles were categorized according to their analysis method and reviewed in each part by their publication date. This helps to identify the trend of works in all the studied methods. First, we review the primary concepts and methods for analyzing EEG inside the MRI scanner presented in the corresponding studies. Then, we cast light on simultaneous EEG-fMRI, elaborating its uses, in both resting state and task completion studies, and measuring the capability of each method in cognitive assessment against other methods. Finally, we address the future perspectives. The current paper tries to strike a balance between task-based studies and those with resting state data. The Preferred Reporting Items for Systematic Reviews and Meta-Analyses (PRISMA) flowchart below demonstrates the organization of the extracted articles (Figure 1). We have strived to include all EEG-fMRI studies focused on functional neurological assessment, which we found through searching for the related keywords in PubMed, Google-scholar, Research-gate, Scopus, and other sources.

(i): Web of science, Google-scholar, Pub Med, Scopus, Research-gate, and the references of the research and review articles; (ii): Not relevant to research questions aims and objectives/or old articles; (iii): Intracranial studies, wrong setting, wrong patient population, wrong intervention, etc.

## Primary concepts

### Electroencephalography

One of the most extensively utilized procedures for studying brain electrical activity is EEG. Berger, who used radio equipment to record brain electrical activity, performed the first electroencephalograph acquisition over 50 years ago. The discovery of EEG, and therefore of brain electrical activity, opened the doors to extensive studies of brain functions, and it became a standard instrument in clinical and research settings over time (Beres, 2017). The electrical activity of the brain is generated by the synchronous activity of a pool of cortical neurons, specifically of pyramidal cells. These cells have a different electrical charge along the neuron, with dendrites being negative and the rest of the cell being positive. This difference creates an electric dipole, which EEG electrodes can detect and display as a series of positive and negative waves. However, a single pyramidal cell’s electric field is insufficient to produce a discernible EEG signal. As a result, the electrodes capture a pool of pyramidal cells that are oriented parallel to one another and produce radial and tangential dipoles (Olejniczak, 2006; Jackson and Bolger, 2014; Nikravan and Ebrahimzadeh, 2021).

**FIGURE 2:**
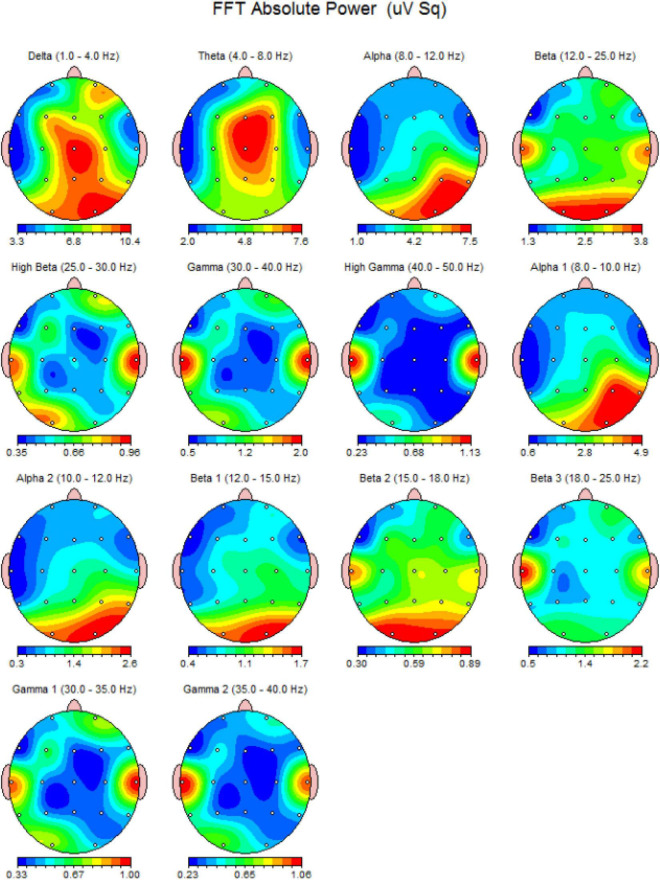
An EEG power spectrum of a 23-year-old healthy volunteer. It presents a topographic representation of each power-band activity in detail. The data obtained from 19-channel MitsarEEG-202 device. The activations are shown in jet color map.

The acquisition of EEG is conducted through the electrode placement on the scalp based on the international 10–20 system which considers four major reference point: inion, nasion, and the two particular points (Klem et al., 1961). The electrodes are attached to the scalp using conductive paste and record a variety of brain oscillations, including delta (0.5–4 Hz), theta (4–8 Hz), alpha (8–13 Hz), beta (13–30 Hz), and gamma (above 30 Hz) (Abreu et al., 2018; Figure 2). Furthermore, evoked potentials can be recorded during task performance, allowing researchers to analyze various neural processes (Sur and Sinha, 2009). The evoked potentials can be divided according to latency. In fact, the potentials that emerge within the first 100 ms after a stimulus are usually attributable to the stimulus nature, whereas the later components reflect the cognitive processes involved in the stimulus perception. For Quantitative EEG (QEEG) and brain connection investigations, novel technologies have led to the development of high-density EEG systems with a large number of channels/electrodes (Astolfi et al., 2007). Currently, EEG is used to characterize a variety of diseases in a clinical setting (configuration with about 20 electrodes), including metabolic or drug alternations, sleep disorders, epileptic syndromes, neurodegenerative diseases, traumatic brain injury, and tumor lesions, as well as the characterization of comatose patients and brain death (Aminoff, 2005).

In a variety of physiological conditions and disorders including Alzheimer’s diseases (AD) (Briels et al., 2020), Multiple Sclerosis (MS) (Seraji et al., 2021), epilepsy (Amoozegar et al., 2013; Gholipour et al., 2022), and depression (Peng et al., 2019), EEG has been used to evaluate brain functional connectivity.

### Functional magnetic resonance imaging

The fMRI modality is one of the most important non-invasive tools for evaluating brain function. The blood oxygen level dependent (BOLD) effect, which defines the variation in the magnetic state of red blood cells according to hemoglobin oxygenation, is the mechanism that subtends the fMRI signal. Indeed, deoxyhemoglobin is paramagnetic whereas oxyhemoglobin is diamagnetic. The balance between the concentrations of these two elements in the vascular system produces a signal that is distinguishable from the surrounding parenchyma in resting conditions (Mele et al., 2019).

Applying a stimulus causes the hemoglobin balance in specific areas of the brain to change, primarily in favor of deoxyhemoglobin concentration, which shows a signal decrease, and if it is in favor of oxyhemoglobin concentration, it shows an increase in the fMRI signal (Buxton, 2002).

Following the execution of specific tasks, the identification of these signal changes results in a series of images that may be studied to indicate the activations of specific brain areas. It is notable that an indirect measurement of neuronal activation is the BOLD effect, which depends on neurovascular coupling and on different interplay, such as alteration in blood flow and volume and complex interactions between the activated neurocircuitry with astrocytic and vascular targets. In the so-called tripartite synapse, neuronal activation induced by the stimulus determines neurotransmitter release in the synaptic cleft and reabsorption (recycling) by the astrocytic process (Jueptner and Weiller, 1995; Menon and Kim, 1999). The production of vasoactive peptides by secondary astrocytic activation causes intracellular Ca^2+^ fluctuations in astrocyte end-feet, which cause cellular biochemical and hemodynamic alterations that are observed by fMRI (Figley and Stroman, 2011).

This complex cascade of events subtends neurovascular coupling and the BOLD effect. It also controls the time delay between neuronal activation and BOLD signal fluctuation that distinguishes fMRI from direct measurement of neural activity.

**FIGURE 3:**
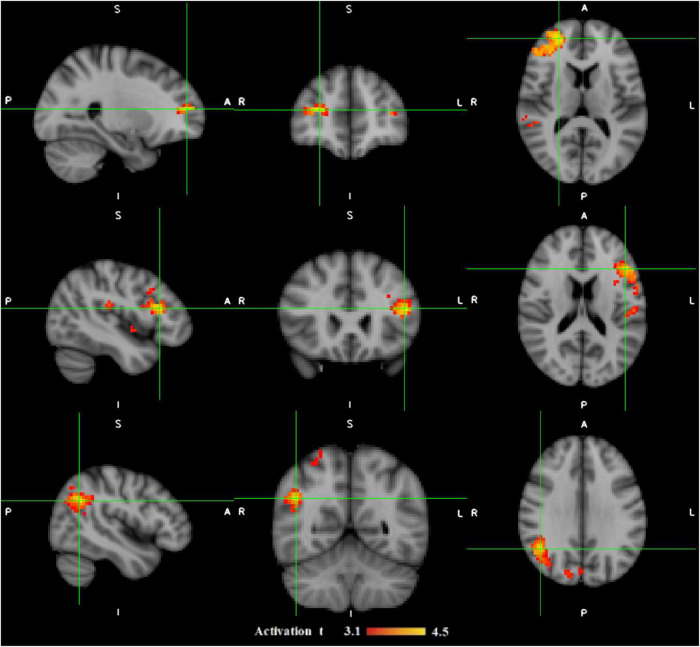
The illustration of three epileptic foci (in each row) for three different patients with refractory focal epilepsy which are localized by functional connectivity analysis on fMRI data. The patient in the top had their seizure focus localized in frontal right; the one in the middle showed epileptic activation originated in fronto-temporal left; and the one in the bottom was predicted to have their epileptic focus in parietal right. The activation regions with *t*-values from 3.1 to 4.5 are shown in red-yellow color map. A, Anterior; I, inferior; L, Left; P, Posterior; R, Right; S, Superior (Sadjadi et al., 2022).

While task-related fMRI has been used in many studies to investigate specific functions and/or brain areas (Ebrahimzadeh et al., 2021a), the resting-state fMRI approach is emerging to analyze spontaneous physiological fluctuations without the need for patient compliance, pathway integrity, or command following, which can be difficult in some patients (Soddu et al., 2011; Cavaliere et al., 2018a). fMRI has been applied to characterize brain functional connectivity in several physiological conditions (Aiello et al., 2015; Marchitelli et al., 2016) and many diseases, including brain tumors (Metwali et al., 2019), MS (Pareto et al., 2018), AD (Liu et al., 2008; Marchitelli et al., 2018), epilepsy (Ebrahimzadeh et al., 2018b,^2019^, 2021a,2021b; Omidvarnia et al., 2019; Sadjadi et al., 2021), and also psychiatric disorders (Mwansisya et al., 2017; Cavaliere et al., 2018b; Figure 3).

## Simultaneous and non-simultaneous functional magnetic resonance imaging and electroencephalography data acquisition

Research on integrated EEG and fMRI can be performed with either non-simultaneous or simultaneous data acquisition. Using the first method, the acquisition of EEG is conducted outside the MR scanner in a separate session, and the sessions are arranged randomly across the patients (George et al., 1995; Menon et al., 1997); while with the second method, fMRI and EEG are recorded inside the MR-scanner (Ebrahimzadeh et al., 2021b; Sadjadi et al., 2021).

### Non-simultaneous electroencephalography-functional magnetic resonance imaging acquisition

**FIGURE 4:**
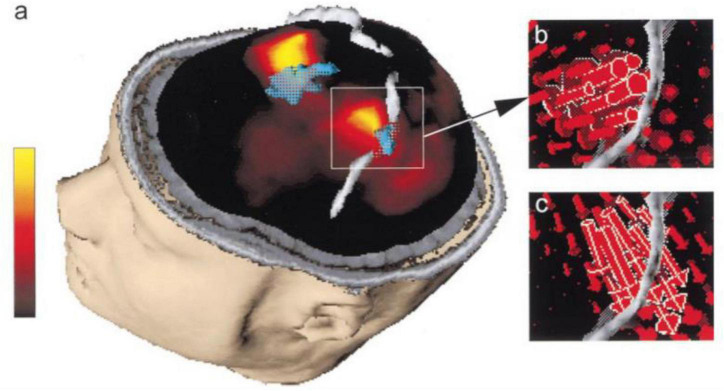
“Primary sensorimotor area activation. **(a)** Rendering of the segmented head of subject 3, view from left and above. The segmented left and right central sulci are shown in gray. Current density map (maximum in yellow, minimum in black, linear scale shown on the left) at EMG onset is shown embedded in the section of the segmented head. fMRI-activated volume is shown in turquoise. The lateral maximum of cortical current density corresponds to the fMRI activation of the hand area of the left primary sensorimotor cortex and the medial current density maximum corresponds to the fMRI activation of the frontal medial wall motor areas. **(b)** Magnification of the hand area of the left primary sensorimotor area as shown in **(a)**. The individual current vectors of the CCD source are displayed, whereas in the current density map in **(a)** only the absolute values of the currents are visualized. In their sum they are equivalent to the current density source at EMG onset shown in **(a)**. The currents point in an anterior direction, consistent with activation of MI in the anterior bank of the central sulcus (Kristeva-Feige et al., 1994). **(c)** Current vectors in the same area as in **(b)** are shown 70 ms after EMG onset. The currents point in a posterior direction, consistent with activation of SI in the posterior bank of the central sulcus (Kristeva-Feige et al., 1994).”(Ball et al., 1999)—reprinted from (Kristeva-Feige et al., 1994) with permission.

Many research projects have integrated EEG and fMRI through data using that is acquired separately in different sessions (Heinze et al., 1994; George et al., 1995; Snyder et al., 1995; Menon et al., 1997; Ball et al., 1999; Opitz et al., 1999). A benefit of separate recording EEG and fMRI is that the signal-to-noise ratio of EEG acquired outside the MR scanner is usually much better than that of the EEG acquired inside the scanner (Figure 4).

Although data are recorded in two separate sessions, differences between the sessions can be minimal, especially those involving ERPs. This is certainly the case for several standard ERP paradigms, such as the oddball (“P300”) and the semantic mismatch (“N400”), in both of which ERPs may, in fact, be better recorded outside the scanner. Techniques for fMRI and EEG acquisition follow standard protocols and therefore, are not mentioned further here. Acquiring EEG and fMRI non-simultaneously has a main drawback (Snyder et al., 1995; Menon et al., 1997). The data of single trial fMRI and EEG cannot be integrated to investigate emergent responses of brain which may not be time locked to the response or stimulus (Heinze et al., 1994).

There also may be major empirical and subjective differences for ERPs between the two separate sessions. For example, the subjects’ level of vigilance, attention, familiarity with the task, and motivation may be different. The data extracted from the simultaneous EEG-fMRI demonstrated that arousal levels are a significant determinant of brain activation over the cognitive tasks (George et al., 1995; Ball et al., 1999).

Moreover, it should be considered that the environment of the two separate sessions are not the same. Unlike fMRI that is acquired in a noisy room, EEG is recorded in a quiet and comfortable environment. This is a notable issue for integrated fMRI and EEG investigations of auditory processing (Snyder et al., 1995).

In the past, when it was not easily possible to have a simultaneous record, most studies would use interleaved EEG–fMRI acquisition protocol, in which EEG data was continuously collected and fMRI data was intermittently acquired. Following stimulus presentation, 1–2 s of EEG data was collected without fMRI scanning (the MRI scanner was silent throughout this time interval), followed by a few seconds of fMRI data collection. This is analogous to the “clustering” procedure employed recording fMRI data in auditory experiments (Hall et al., 1999), where auditory stimuli must be heard without interference from scanner noise. Because the neurophysiological response begins a few milliseconds after the stimulus initiation and lasts no more than 1–2 s, depending on the cognitive processes involved in the task, the majority of the valuable stimulus-related EEG is not contaminated by scanner noise.

### Simultaneous electroencephalography-functional magnetic resonance imaging

The correlation between hemodynamic mutation and electrical brain activity is evaluated using EEG-fMRI data. Due to the slow BOLD response (in the order of seconds), fMRI with high spatial resolution does not provide enough temporal sampling, but EEG does (in the order of milliseconds), albeit with poor signal source localization (Iannaccone et al., 2015; Ebrahimzadeh et al., 2018b).

**FIGURE 5:**
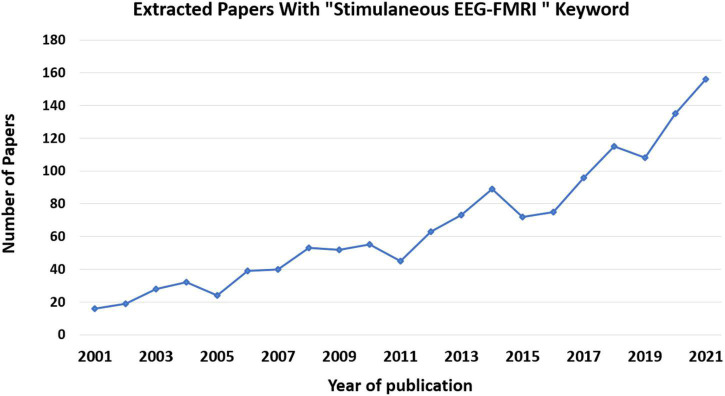
Number of papers published per year on simultaneous EEG-fMRI. These data came from Pubmed site by searching for “Simultaneous EEG-fMRI” in Title/Abstract.

Among the available imaging modalities, analysis of fMRI and EEG data and the simultaneous recording is considered to be the cutting-edge multimodal imaging method and has received considerable attention (Herrmann and Debener, 2008; Huster et al., 2012; Manganas and Bourbakis, 2017; Mele et al., 2019). This notion can be substantiated by the large number of related scientific papers published in this field since 2000, as illustrated in Figure 5.

Through the integration of these two techniques in a hybrid simultaneous acquisition, it would be possible to overcome the limitations of each modality. It also increases the plethora of analyses that can be conducted, and in turns, of the data that can be obtained (Marawar et al., 2017). Simultaneous acquisition also guarantees an identical registration, as regards the mental state of the subject, the execution of the task and the inference of the recording environment (Fleury et al., 2019).

Acquisition of EEG-fMRI simultaneously, also allows for an identical registration, with respect to the mental state of the subject, the task execution and the acquisition environment inference. This does not happen by recording the two methods separately, especially if the recording takes place in different environments from cognitively unstable patients (Debener et al., 2006).

Unlike simultaneous acquisition, recording the two modalities separately does not provide identical registration, especially if the acquisition takes place in different environments and involving patients with cognitive instability. As for technological issues, simultaneous EEG/fMRI acquisition involves the use of specialized EEG hardware that is safe and compatible with the MRI environment and comfortable for the subject. Using the equipment improperly chosen may result in considerable risks. Concerning safety, there is a potential risk for the subjects coming from electrodes and heating of conducting leads during MR radio frequency transmission, resulting in discomfort or even burns (Kugel et al., 2003). There are several precautions in order to reduce the risk of participant discomfort or injuries, for instance, fMRI sequences should be based on gradient echo-echo planar imaging (GE-EPI); for anatomical reference scans, low specific absorption rate (SAR) sequences, particularly GE-T1 weighted sequences, should be used; and for all sequences in the EEG-fMRI protocol, the SAR should not exceed the SAR of the GE-EPI sequence. Otherwise, it is necessary to perform extensive safety testing with temperature sensors. Staff performing EEG-MRI studies must have received appropriate training, as injuries due to MR-compatible EEG equipment cannot be ruled out if the equipment is accidentally used out of specifications, especially in the case of body coil transmission (Nöth et al., 2012). The adoption of these guidelines is especially important in vigilance-reduced subjects (sleeping or sedated subjects) or, generally, in subjects who cannot give notice of any discomfort reliably, such as children. When employing EEG-fMRI, it is critical to ensure that the participants have a thorough comprehension of all processes involved, that they are comfortable with all steps, and that there are no accidents that could cause discomfort, resulting in movement and, consequently, failure of the experiment (Cavaliere et al., 2018a). Participants should be aware that nothing will be painful, even if some stages may be mildly unpleasant, such as minor scalp abrasion during EEG electrodes setup; this improves the participant’s calmness which is necessary for the experiment to be completed successfully and securely.

**FIGURE 6:**
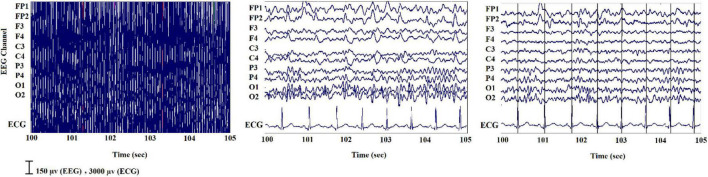
A sample of EEG signals recorded inside the scanner: (Left) primary signal; (Middle) after eliminating the MR gradient artifact; (Right) after removing the BCG artifact (Ebrahimzadeh et al., 2021b).

Artifacts greatly influence the data obtained from simultaneous EEG-fMRI (Scrivener and Reader, 2022). On one hand, wearing the helmet causes a variation in the magnetic field’s homogeneity, resulting in a variation in image quality; on the other hand, the magnetic field itself causes broad-band artifacts that almost entirely cover the electroencephalographic signal (Steyrl and Müller-Putz, 2019). Another artifact that is observed on the EEG recorded inside the scanner is the ballistocardiogram (BCG) artifact originated from electrodes movement caused by pulse related in the static magnetic field that is influenced by the spatio-temporal variability of cardiac cycles during recording (Figure 6; Marino et al., 2018).

**FIGURE 7:**
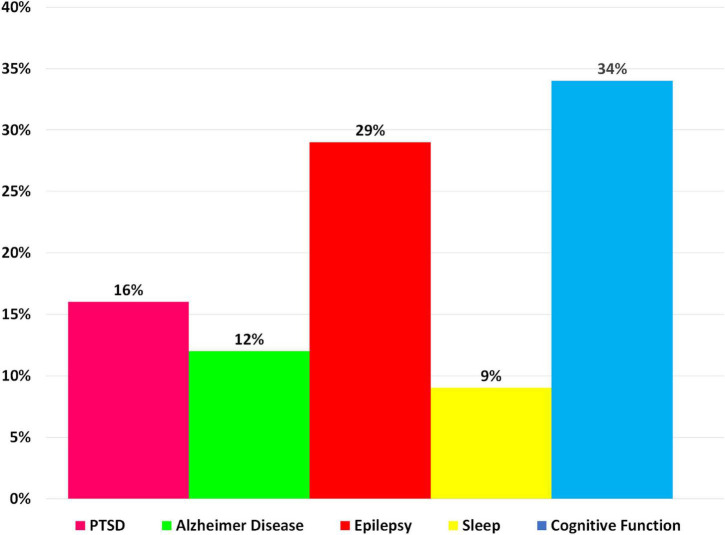
A bar chart illustrating the proportion of simultaneous EEG-fMRI studies in relation to neuropsychological impairments compared with each other in a way that shows the contribution of each neurological disorder in EEG-fMRI studies.

Researchers have devised a variety of artifact removal techniques, including independent component analysis (ICA), which is widely regarded as the best method for removing the BCG artifact (Srivastava et al., 2005). In order to cover studies with simultaneous acquisitions of EEG-fMRI both during task performance and in resting state, we have collected scientific literature published since 2014 on the PubMed website using the key word “Simultaneous EEG-fMRI” (Figure 7). In Figure 7, the cognitive functioning refers to multiple mental abilities, including learning, thinking, reasoning, remembering, problem solving, decision making, and attention.

### Simultaneous resting-state electroencephalography-functional magnetic resonance imaging

Even while in a state of rest, the brain is a dynamic mechanism that generates activity. This can be demonstrated by fMRI through the estimation of different resting state networks linked to specific cerebral functions and by EEG recording through the detection of varied frequency and amplitude neural waves. The simultaneous recording of rsfMRI and EEG allows researchers to think of the brain as a collection of interconnected networks or systems (Deligianni et al., 2014; Tsuchimoto et al., 2017). The interactions are influenced by the brain’s electrical activities as well as the concurrent variation of BOLD fluctuations. The simultaneous recording of EEG and rsfMRI data has a wide range of applications. Table 1 refers to a summary of the resting state EEG-fMRI studies since 2014. Seven different application areas are presented to cover an acceptable range.

**TABLE 1:** A summary of the resting state EEG-fMRI studies since 2014.

References	Application field	Subjects	MR field	MR sequence	EEG	Analysis	Results
Brueggen et al. (2017)	Alzheimer	14 AD patients 14 healthy controls	3T	EPI sequence TR = 2.5 s TE = 30 ms RES: 3.5 × 3.5 × 3.5 mm	Brain products 32-channel	Voxel-wise linear regression analyses with the regressor of power within the EEG alpha band	Diminished positive association between the BOLD signal fluctuations and alpha band power fluctuations in several brain regions of AD patients in comparison with the healthy controls.
Dong et al. (2016)	JME	18 JME patients	3T	EPI sequence TR = 52,000 ms TE = 30 ms RES: 1 × 1 × 1 mm	Neuroscan 62-channel	Eigenspace maximal information canonical correlation analysis (emiCCA) and functional network connectivity (FNC)	Linear and non-linear relationships between discharge-affecting networks and epileptic discharge information in JME patients
Deligianni et al. (2014)	Connectivity	17 Healthy subjects	1.5T	EPI sequence TR = 2,160 ms TE = 30 ms RES: 3.3 × 3.3 × 4.0 mm	Electrode cap (BrainCap MR) 64-channel	Relating the covariance matrices based on sCCA	Correlation between the EEG signals and their anatomical zones of generation.
Marawar et al. (2017)	Sleep	14 Healthy sleep-deprived subjects	3T	EPI sequence TR = 2,000 ms TE = 30 ms RES: 4 × 4 × 4 mm	fEEG; Kappametrics Inc., Chantilly, VA	GLM analysis using the time series of EEG delta and theta band power corresponding to bilateral temporal lobes	Different correlations for the delta and theta rhythms.
Keinänen et al. (2018)	Epilepsy	10 DRE patients 10 healthy controls	3T	MREG TR = 100 ms TE = 35 ms RES: 4.5 × 4.5 × 4.5 mm	BrainAmp 32-channel	Short-time-window comparisons of infra-slow full-band EEG and BOLD signals	There is a role of intrinsic brain pulsations in DRE which can be identified by critically sampled fMRI.
Yuan et al. (2018)	PTSD	36 PTSD patients 20 combat-exposed (controls)	3T	EPI sequence TR = 2,000 ms TE = 30 ms RES: 1.875 × 1.875 × 2.9 mm	Brain products (BrainAmp MR Plus amplifiers) 32-channel	Integrating EEG and fMRI by quantifying fast temporal dynamics related to the resting-state networks	Correspondence between the temporal dynamics of DMN and PTSD severity.
Yin et al. (2016)	Motor control	36 Healthy subjects	3T	EPI sequence TR = 1,980 ms TE = 30 ms RES: 3.5 × 3.5 × 3.5 mm	Brain products (MR-compatible) 32-channel	Correlation between power fluctuations of mu components convolved with canonical HRF and BOLD signals.	Positive correlation between the power of Mu rhythms and BOLD within the anterior cingulate cortex and the anterior insula.

BOLD, Blood-Oxygen-Level-Dependent; DMN, Default Mode Network; DRE, Drug-resistant Epilepsy; GLM, General Linear Model; HRF, Hemodynamic Response Function; ICA, Independent Component Analysis; JME, Juvenile Myoclonic Epilepsy; MREG, Magnetic Resonance Encephalography; PFC, Prefrontal Cortex; PTSD, Posttraumatic Stress Disorder; sCCA, sparse Canonical Correlation Analysis.

The first research looked at methodological concerns in healthy people, investigating the reconstruction of EEG signal sources using fMRI data and primarily focusing on connectivity analyses. However, in order for the theory to be validated, the study sample is necessary (Deligianni et al., 2014). In seventeen adult volunteers, the authors demonstrated that a simultaneous approach using a 64 channel MRI-compatible EEG cap was useful in validating whole-brain connectomes generated by each method and developing a prediction model of dynamic functional connectivity (Deligianni et al., 2014).

Another study (Marawar et al., 2017) correlated temporal lobe theta and delta EEG frequencies and simultaneous fMRI acquisition in fourteen healthy sleep-deprived adults in drowsy and awake states. The recent study discovered a new brain regional source for theta and delta frequencies for the first time, while they also looked at the fastest frequencies like alpha, beta, and gamma (Marawar et al., 2017). This type of approach decreases the localization of the sources generating different EEG bands but has an advantage of producing a greater differentiation of the slow frequencies. The electrical BOLD correlation appeared to be larger for frequencies below 1 Hz, and is influenced by the spatial relationship between the investigated resting state networks and recording zones (Keinänen et al., 2018).

**FIGURE 8:**
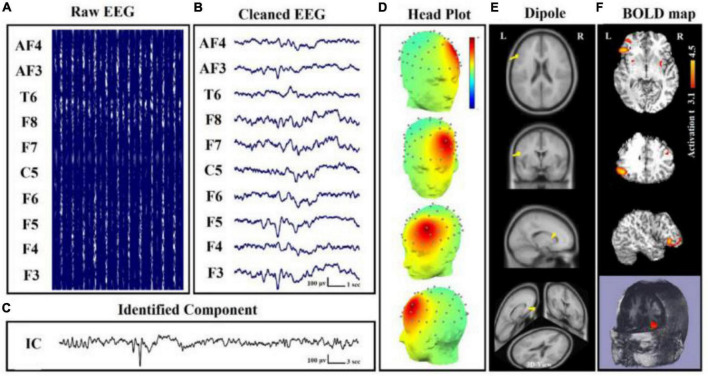
Simultaneous EEG-fMRI analysis on a patient with focal epilepsy in order to localize the seizure onset zone (SOZ). **(A)** Simultaneously recorded raw EEG data. **(B)** The EEG data after artifact removal. **(C)** The epilepsy-related component time series obtained using Independent Component Analysis (ICA) algorithm. **(D)** The 3D map of the candidate component. The active area is marked by yellow-red color. **(E)** The result of dipole localization of the identified generator in deep brain structures. **(F)** The final result of localizing the SOZ applying simultaneous analysis of EEG–fMRI (Ebrahimzadeh et al., 2019, 2019a).

The basis of some specific electrical oscillations, such as the mu frequency, has also been studied using this relationship. EEG-fMRI was used to identify a positive correlation between the strength of mu frequency and the BOLD signal in regions such as the anterior insula and the anterior cingulate cortex in a research of 36 healthy people, confirming the numerous origins of this specific frequency (Yin et al., 2016). In terms of neurological illnesses, a study on 18 individuals with juvenile myoclonic epilepsy demonstrated the utility of EEG-fMRI acquisition in revealing the disease’s pathogenesis, with a focus on the relationship between frontal networks and epileptic discharges (Dong et al., 2016). Authors of Brueggen et al. (2017) found a lower correlation between occipital alpha band power and BOLD signal fluctuation in the frontal and temporal cortices, as well as the thalami, in fourteen Alzheimer’s patients. Other researchers in psychiatry discovered a connection between the temporal dynamics of the default mode network and the severity of post-traumatic stress disorder (PTSD) in 36 veterans compared to twenty combat-exposed controls (Yuan et al., 2018). It has become clear that simultaneous EEG-fMRI can provide significant information on the relationships between neuronal activity and BOLD response, and that the resting state acquisition, in particular, can be crucial for understanding the variability of brain activity and, more importantly, for identifying the structures involved in triggering EEG waves in the resting state (Figure 8). In this context, expanding the sample size and using different methods of analysis can help to corroborate prior findings and disentangle controversial or inconsistent findings.

### Simultaneous task electroencephalography-functional magnetic resonance imaging

**FIGURE 9:**
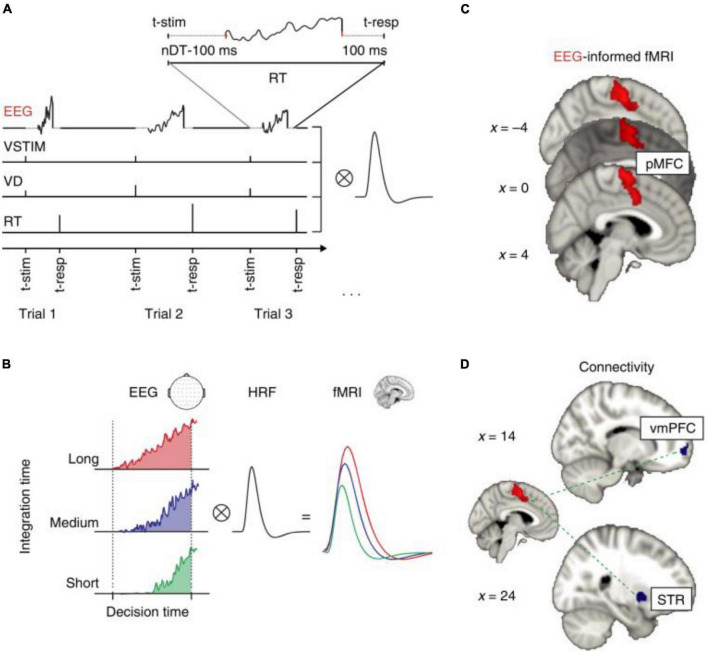
“EEG-informed fMRI and connectivity analyses in the value-based task. **(A)** The fMRI GLM model included an EEG-informed regressor capturing the electrophysiological trial-by-trial dynamics of the process of evidence accumulation (EA) in each participant. Three actual single-trial EEG traces are shown. The traces cover the entire trial excluding the time intervals accounting for stimulus processing and motor execution (see inset and Methods for details). To absorb the variance associated with other task-related processes we included three additional regressors: VSTIM—an unmodulated stick function regressor at the onset of the stimuli, VD—a stick function regressor at the onset of stimuli that was parametrically modulated by the value difference between the decision alternatives and RT—a stick function regressor aligned at the time of response and modulated by RT. **(B)** Hypothetical EA traces in response-locked EEG activity ramping up with different accumulation rates. Convolving these traces with a hemodynamic response function (HRF) leads to higher predicted fMRI activity for longer compared to shorter integration times (that is, the predicted fMRI response scales with the area under each EA trace). **(C)** The EEG-informed fMRI predictor of the process of EA revealed an activation in pMFC. **(D)** PPI analysis using the pMFC cluster identified in c as a seed revealed an inverse coupling with a region of the vmPFC and the STR. All activations represent mixed-effects and are rendered on the standard MNI brain at |Z| 42.57, cluster-corrected using a resampling procedure (minimum cluster size, 76 voxels)” (Pisauro et al., 2017).

Researchers can determine which cerebral areas are assigned to specific activities based on the cognitive domain investigated by executing tasks. Simultaneous EEG-fMRI tests using a recognition memory task have shown that theta-alpha low frequency oscillations (4–13 Hz) are connected with the functional activation of a network involving the striatum, prefrontal cortex, and hippocampus. This finding is backed with the hypothesis that the hippocampus works as a brain activity modulator via low frequency oscillations (Herweg et al., 2016). The hippocampus appears to play an important role during sleep as well. In fact, studies have shown that hippocampal activity rises during light sleep and correlates with alpha activity (Knaut et al., 2019). This observation could support the hypothesis that memory consolidation can occur during light sleep stages (Knaut et al., 2019). A simultaneous method has been used to explore neural foundations for perceptual decisions and evidence collection in decision making evaluation, indicating a common involvement for the posterior medial frontal cortex in both processes (Figure 9; Pisauro et al., 2017). The execution of tasks allows to establish, according to the cognitive domain studied, which cerebral areas are assigned to each specific task (Table 2). Since there is a large literature in this field, we have clustered the articles based on different tasks and from each cluster we have displayed one representative published in recent years in Table 2.

**TABLE 2:** A summary of the task EEG-fMRI studies since 2014.

References	Application field	Subjects	MR field	MR sequence	EEG	Task	Results
Herweg et al. (2016)	Behavioral/cognitive	19 healthy controls	3T	EPI sequence TR/TE = 4,000/25 ms RES: 2 × 2 × 2 mm	Brain products (Braincap MR) 64-channel	Recognition memory task inside the MR scanner.	There is a link between theta-alpha power and hippocampal connectivity with the striatum and PFC.
Zotev et al. (2018b)	Neurofeedback	15 healthy controls	3T	EPI sequence TR/TE = 5.0/1.9 ms RES: 0.94 × 0.94 × 1.2 mm	Brain products 32-channel	Retrieval of happy autobiographical memories	Emotional control training can make an improvement in alpha-band activity and functional connectivity of prefrontal cortex and amygdala.
Pisauro et al. (2017)	Neuroscience	21 healthy controls	3T	EPI sequence TR/TE = 2.5 s/40 ms RES: 3 × 3 × 3 mm	Brain Amps (MR-Plus) 64-channel	Independent reward-based decision-making task	Task-dependent correlation with the ventromedial prefrontal cortex and the striatum.
Andreou et al. (2017)	Translational psychiatry	22 healthy controls	3T	EPI sequence TR/TE = 2,000/25 ms RES: 1 × 1 × 1 mm	BrainVision 64-channel	Gambling task	Positive feedback: Rise in beta power reflecting the activation of subcortical areas. Negative feedback: rise in theta power associated with activation of fronto-parietal areas.
Guo et al. (2017)	Neuropsychology	20 healthy controls	3T	EPI sequence TR/TE = 2,000 ms/35 ms RES: 1 × 1 × 1 mm	Net station 64-channel	Monetary gambling task	EEG-fMRI acquisition during gambling task underline activation of a posterior cingulate, ventral striatum, and medial prefrontal cortex.
Zotev et al. (2018b)	Neurofeedback	30 PTSD patients	3T	EPI sequence TR/TE = 2,000/30 ms RES: 1.875 × 1.875 × 2.9 mm	Brain products 32-channel	Thinking of and writing down five happy autobiographical memories	The potential of rtfMRI-nf of the amygdala activity in correcting the amygdala-prefrontal functional connectivity deficiencies specific to PTSD.
Zich et al. (2015)	Neuropsychology	24 Healthy controls	3T	EPI sequence TR/TE = 1.5 s/2.52 ms RES: 3.1 × 3.1 × 3.0 mm	Brain products 32-channel	EEG neuro-feedback motor task	MI EEG signals showed a complex relationship with sensorimotor cortical activity and this supports the role of MI EEG feedback in motor rehabilitation.

PFC, Prefrontal Cortex; PTSD, Posttraumatic Stress Disorder.

It has been demonstrated that an increase in theta band power associated with a choice with negative feedback corresponds to the activation of fronto-parietal areas; on the other hand, an increase in the power of the beta band associated with positive feedback corresponds to the activation of subcortical areas involved in the reward network (Andreou et al., 2017). Using a gambling task paradigm, concurrent activation of significant areas connected to reward and punishment, such as the ventral stratum, posterior cingulate, and medial prefrontal cortex, has been shown (Guo et al., 2017).

Neurofeedback is another use of EEG-fMRI, which allows for the modulation of brain processes, despite the fact that the information returned to the patient so far has only been EEG (Zich et al., 2015) or fMRI related (Guo et al., 2017; Zotev et al., 2018a,b). Several scientists have studied brain activation during movement execution and motion imaginations in healthy volunteers using EEG neurofeedback, suggesting that this approach could be useful in the rehabilitation of patients with post-stroke paralysis (Zich et al., 2015). In terms of fMRI neurofeedback, two studies have looked into the relationship between the BOLD signal and the EEG signal after behavioral modulation. The first study found that modulating thalamic nuclei activation during retrieval of pleasant autobiographical memories can affect both the alpha frequency and the BOLD signal in a group of 34 healthy people (Zotev et al., 2018a). The second study, which was conducted by the same researchers in a group of PTSD patients, found that emotional control training improved alpha frequency and functional connectivity between the prefrontal cortex and the amygdala, and that this improvement was correlated to improved clinical performance (Zotev et al., 2018b). Only one publication has so far described the use of a novel simultaneous real-time fMRI and EEG neurofeedback system (Zotev et al., 2014). The authors demonstrated that emotional self-regulation training based on recall of joyful autobiographical experiences could modulate both BOLD fMRI activation of the amygdala and beta band EEG power asymmetry in healthy subjects (Zotev et al., 2014).

To summarize, task EEG-fMRI provides significant evidence for cognitive and affective processes. This is an excellent place to start learning about and discovering everything there is to know about mental and neurological diseases that are still a mystery. Despite the fact that the multimodal approach identifies several issues that can complicate the research process, simultaneous EEG-fMRI acquisition remains one of the most popular approaches, as it allows for a comprehensive view of brain activity while minimizing the impact on the subjects involved in the study.

## Clinical applications in integrating functional magnetic resonance imaging and electroencephalography

There is growing interest in combining single trial, or non-phase-locked, EEG with fMRI to evaluate the clinical applications. Integrating EEG and fMRI or simultaneous EEG-fMRI recording makes it possible to look at a phenomenon from different perspectives (Mulert and Lemieux, 2009). Each of these approaches represents some of the hidden dimensions of reality in clinical studies (Moeller et al., 2020). These signals are related to perceptual and cognitive processing, but they may or may not be locked to an external stimulus. Combined EEG–fMRI methods have been used to identify the neural correlates of clinically or behaviorally important spontaneous EEG activity, such as interictal spikes (Sadjadi et al., 2022), the alpha rhythm, and sleep waves. The area where great progress has been made is epileptic-seizure localization. The common method of integrating EEG and fMRI data here is to transform EEG data into a physiologically meaningful covariate to be used in a voxel-based general linear model. The EEG-fMRI combined method improves and contributes to the literature through substantially increasing the unimodal studies yield to localize the epileptic foci in a non-invasive manner, enabling a more thorough and reliable evaluation prior to surgery, especially in patients with refractory epilepsy (Ebrahimzadeh et al., 2018b,^2019^, 2019a,2021b), or placement of intracranial electrodes (Tehrani et al., 2021). This allows for providing more patients with the option of surgery while increasing the likelihood of a successful and life-improving operation (Ebrahimzadeh et al., 2019b).

### Epileptic focus localization

The ability to record EEG during fMRI has opened up new possibilities for epilepsy research. Until recently, the earliest uses of single-trial EEG and fMRI were limited to seizure localization. On scalp EEG, interictal discharges underlying epileptogenic neuronal activity can be easily seen; nevertheless, the source of this activity can only be inferred in terms of lobes and hemispheres. The epileptic focus localization is relatively different from other EEG–fMRI applications since each patient’s epileptogenic activity is distributed differently spatially and temporally, and signatures of this activity must be determined with a high degree of precision and efficiency (Beers et al., 2015; Ebrahimzadeh et al., 2019, 2019a,2019b,2021b).

Several groups have employed simultaneous EEG and fMRI measurements to study interictal activity in individuals with epilepsy in recent years (Krakow et al., 1999; Lemieux et al., 2001; Aghakhani et al., 2004; Federico et al., 2005; Pittau et al., 2012; An et al., 2015). Although early research (Krakow et al., 1999; Pittau et al., 2012; An et al., 2015) employed EEG spikes to trigger fMRI acquisition, it is now typical to use continuous EEG and fMRI data (Rosenkranz and Lemieux, 2010). In either case, the comparatively large amplitude of epileptogenic activity, which is detectable on scalp EEG at around 100 μV, has aided these studies. This is as opposed to perceptual and cognitive ERPs, which have amplitudes of about 5–10 μV after averaging over several trials; although in some people with epilepsy, the events are in fact infrequent and of short duration [from a fraction of a second to (rarely) more than 10 s] (Bagshaw et al., 2006). In these cases, studying interictal epileptiform episodes is extremely difficult. The localization of task-correlated language and memory function, as well as the localization of ictal and paroxysmal phenomena, are two major applications of fMRI in epilepsy (Detre, 2004). For example, research from a number of labs has revealed that fMRI language lateralization produces results that are equivalent to intracarotid amobarbital testing (Waites et al., 2005). Recent research has also revealed that generalized spike and wave (GSW) or polyspike and wave bursts in patients with idiopathic generalized epilepsy are caused by extensive regions of the cerebral cortex and thalamus (Aghakhani et al., 2004). Interestingly, both activation and deactivation were observed in relation to the GSW; in the thalamus, activation predominated over deactivation, whereas in the cerebral cortex, the converse was observed. Most human patients have a thalamic BOLD response, which matches the thalamic involvement reported in animal models. These findings further assure the practicality of the combined EEG-fMRI approach for better understanding of the brain networks underlying various types of epilepsy (Lemieux, 2004).

### Advantages and other clinical applications

The fundamental benefit of gathering EEG and fMRI data in the scanner is that they both reflect the same neural processes. Simultaneous acquisition ensures that people employ the same strategy for both types of input; this is especially crucial for activities that require complicated cognitive processing. Simultaneous EEG and fMRI are clearly helpful for epileptic seizure localization (Acharjee et al., 2015; Ebrahimzadeh et al., 2019, 2019a,2019b; Ebrahimzadeh et al., 2021b). Other essential clinical concerns necessitate simultaneous EEG and fMRI, particularly in cases where symptoms fluctuate over short periods of time. During hallucinating periods, for example, some people with schizophrenia have difficulty separating self-produced percepts from externally generated ones, which might wax and wane unpredictably (Scrivener, 2021).

Simultaneous EEG and fMRI recording is becoming increasingly helpful outside of the therapeutic realm. It aids studies of single-trial EEG and fMRI in which researchers want to better understand neural processing that is not always time bound to external events (Makeig et al., 2002; Ahmad et al., 2015). Simultaneous EEG and fMRI recordings are widely used in studies of the resting state and attempts to better understand brain dynamics underpinning intrinsic EEG rhythms (Goldman et al., 2002) and the default mode of brain operation (Laufs et al., 2003a,b, 2006; Raichle and Snyder, 2007).

Simultaneous acquisition is preferred in clinical and developmental studies since it reduces the overall amount of time required to collect data. It is also required in studies where continuous EEG tracks distinct stages of sleep (e.g., REM/non-REM) (Czisch et al., 2004). Simultaneous recordings may be important for practical reasons (Bridwell and Calhoun, 2019). Non-simultaneous recordings may not be feasible in clinical studies, as well as studies of youngsters and the elderly. The majority of these individuals are unwilling to participate in multiple sessions over long periods of time—we’ve seen this across a variety of subject groups. Furthermore, conducting trials across numerous sessions may not be possible, reliable, or practical in clinical investigations involving medicine. Thus, while normative studies on college-aged individuals can readily be completed in two or more sessions, clinical and developmental studies are far more challenging. Memory and learning paradigms can include extended stimulus exposure, which can interfere with encoding and retrieval, even in normal healthy persons, particularly in children.

This is all why several brain-imaging facilities have developed and refined approaches for simultaneous acquisition of EEG and fMRI data (Bonmassar et al., 1999, 2001b; Krakow et al., 1999; Lemieux et al., 2001; Laufs et al., 2003b; Salek-Haddadi et al., 2003; Yu et al., 2016) and used them to detect EEG spikes, define resting-state EEG, and examine the neurological basis of ERPs (Bonmassar et al., 1999, 2001a,2001b,^2002^; Lazeyras et al., 2001; Christmann et al., 2002; Liebenthal et al., 2003; Thees et al., 2003; Mulert et al., 2004; Nagai et al., 2004; Mulert and Lemieux, 2009; Mulert, 2022) over the last years.

### Characterization of structure and dynamics of brain networks

Oscillation patterns in distinct frequency bands carry out functional coupling in the brain, which may be directly measured using methods like EEG and magnetoencephalography (MEG) (Buzsáki and Watson, 2022). For example, in the visual domain (Fisch et al., 2009) or for cognitive processes (Herrmann et al., 2010), high-frequency oscillations in the gamma band (> 30 Hz) play a significant role in the coupling of neuronal ensembles during perceptual processing. Other frequency bands, such as theta oscillations (4–8 Hz), have been found to be crucial for hippocampus-prefrontal cortex communication (Colgin, 2011; Fujisawa and Buzsáki, 2011). Improved understanding of the causes of disrupted connectivity in the brains of mental patients could lead to new therapeutic strategies: disrupted gamma band oscillations, for example, are a typical finding in schizophrenia (Spencer et al., 2003; Leicht et al., 2010).

**FIGURE 10:**
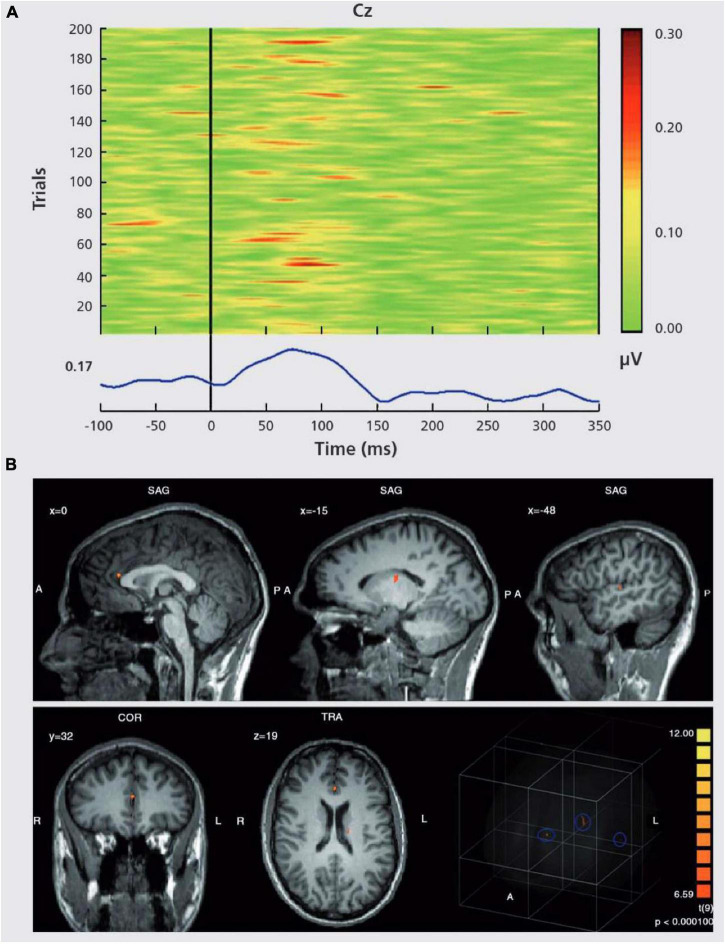
“EEG-informed fMRI analysis in an auditory choice reaction task.” **(A)** EEG single-trial data of a typical data set at electrode Cz. In many trials, a GBR between 30 and 100 ms post-stimulus is present. However, the amplitudes of the GBR are variable over time. This variability can be used for specific predictions of the related BOLD signal. In the lower part of the figure, the corresponding averaged GBR is shown. **(B)** GBR-specific BOLD activation based on the single-trial coupling of GBR amplitude variation and the corresponding BOLD activation. Activations can be seen in the left auditory cortex (gyrus temporalis superior, Brodmann area 41/22, highest *t*-value 8.3), the thalamus (highest *t*-value 7.6) and the anterior cingulate cortex (Brodmann area 24, highest *t*-value 10.1). In the lower right corner, a glass brain view is provided, demonstrating a 3D view of the three abovementioned clusters. Activations are shown at P. (Mulert et al., 2010).

The intact function of the N-Methyl-D-aspartate (NMDA) receptor is required for gamma-band oscillations (Carlen et al., 2012). Improvement of glutamatergic neurotransmission at the NMDA receptor has been proposed as a viable new therapy method for schizophrenia patients (Javitt, 2012). EEG-fMRI can be utilized to describe network topology and relevant coupling mechanisms such as gamma oscillations during cognitive processing (Figure 10; Mulert et al., 2010). As a result, it is crucial to characterize both the network topology and the neurophysiological mechanisms involved, and simultaneous EEG-fMRI provides the methodological foundation for this (Debener et al., 2006; Mulert and Lemieux, 2009).

### Brain oscillations and networks

Several intrinsic brain networks, such as the default-mode network (DMN), the dorsal attention network (DAN), and the salience network, have been found based on the spatial patterns of correlated time series that are reliably recognized in resting state BOLD signals (SN). In time periods of seconds to minutes, brain areas within these networks demonstrate increasing functional connectivity. Several neuropsychiatric diseases, such as Alzheimer’s disease, are associated with changes in resting state networks (Franciotti et al., 2013).

Early resting-state EEG-fMRI research attempted to identify BOLD correlations of certain frequency patterns like alpha oscillations, which will further be investigated in next section (Goldman et al., 2002; Laufs et al., 2003a; Moosmann et al., 2003). However, in order to define network dynamics, it was proposed that the EEG signal be linked to functional connectivity within and between networks. Hlinka et al. (2010) for example, found that delta and beta oscillations account for 70% of the DMN variance in functional connectivity.

When alpha power increases, BOLD connectivity between the primary visual cortex and occipital regions decreases, as does negative coupling between visual areas and DMN regions, according to Scheeringa et al. (2012).

Chang et al. (2013) looked at the DMN, DAN, and SN’s functional connectivity. The connectivity between DMN and DAN was discovered to be inversely related to alpha power. Furthermore, the spatial extent of anticorrelation between DMN and DAN was related to alpha power.

While these studies were conducted with the goal of connecting known fMRI resting state networks and investigating the relationship between EEG power of different frequency bands, another method is to link fMRI patterns with more sophisticated EEG organization patterns. The topographic representation of the EEG, for example, remains consistent during durations of roughly 100 ms. The term “microstates” was used to describe these quasi-stable, one-of-a-kind distributions (Lehmann et al., 1987).

Microstates are the sum of concurrent neuronal activity throughout brain areas, rather than activity restricted to a single frequency range. Microstate changes have been observed in a variety of psychiatric illnesses, including schizophrenia (Lehmann et al., 2005). Several publications have recently described the link between EEG microstates and BOLD resting-state networks using simultaneous EEG-fMRI (Huang et al., 1996; Britz et al., 2010; Musso et al., 2010). The study of the relationship between EEG coherence patterns and fMRI connectivity will be another fascinating approach. The link between interareal BOLD correlations and brain oscillations was recently studied in monkeys using the combination of fMRI and invasive electrophysiological measures (Wang et al., 2012). Although gamma oscillations (30–100 Hz) have previously been demonstrated to be notably firmly connected to the BOLD signal, coherence in low frequencies (20 Hz) contributed primarily to fMRI connectivity patterns in the task-free state. Simultaneous EEG-fMRI and EEG source analysis in humans may be used in the future to do comparable analyses incorporating EEG coherence analyses with fMRI connectivity analyses.

EEG-fMRI is now commonly conducted in many MRI centers after several years of development, and both safety and signal quality issues are well addressed (Mulert, 2022). As mentioned earlier the fact that both EEG and fMRI have overlapping sensitivity to synaptic processing means that brain function can be measured using simultaneous EEG-fMRI with high temporal and spatial resolution. The characterization of brain network structure and dynamics is one of the most promising applications for EEG-fMRI nowadays.

### Relationships between alpha activity and functional magnetic resonance imaging response

EEG-fMRI studies primarily focused on fMRI responses in relation to EEG alpha band (e.g., 8–12 Hz). This was because alpha activity, which can be detected by an untrained experimenter in unprocessed EEG, had continued to appear in individual recordings. The presence of alpha activity is especially important in concurrent EEG-fMRI as the scanner environment can cause significant artifacts in EEG (for review see Moosmann et al., 2003; Thees et al., 2003). The origins and characteristics of the EEG alpha band attracted a lot of research attention over the decades. It was therefore reasonable to place the prime focus of EEG-fMRI studies on alpha activity (Bridwell and Calhoun, 2019).

Alpha oscillations tend to appear at occipital electrodes and show an increase once individuals close their eyes, are sleepy, or engage in mental arithmetic (Klimesch et al., 2007). Since such tasks involve a lesser degree of visual cortical activity, increased occipital alpha activity is associated with cortical inactivity. This inactivity limits the ability of visual areas to affect areas of the brain that are responsible for current cognitions or tasks. For instance, increases in alpha correspond to decreased resting-state connectivity between early visual areas and the rest of the brain (Scheeringa et al., 2012). Increased visual inactivity is also interpreted as increased synchrony across visual areas, increased dependence across areas, and an overall reduction in visual complexity (Patel et al., 2015). These processes could also indicate reduced cortical metabolism. Since BOLD fMRI is sensitive to metabolic processes, this theory has been put to a test: EEG-fMRI studies have shown negative relationships between alpha activity and occipital, parietal, temporal, and fontal fMRI responses, and positive correlations between alpha and the thalamus (Goldman et al., 2002; Liston et al., 2006; Bridwell and Calhoun, 2019). The negative relationship is in line with the idea that increased alpha activity mirrors reduced cortical metabolism and a subsequent reduction in the BOLD fMRI response. Also, increased metabolism reflects increased fMRI responses and a reduction in alpha. Moosmann et al. (2003) went on to provide more proof by finding a negative relationship between changes in deoxy hemoglobin [measured by near infrared spectroscopy (NIRS)] and alpha EEG. The majority of studies also confirm a negative correlation between fMRI and EEG alpha activity; therefore, it is regarded as one of the reliable and consistent results in the literature. It can serve as a useful “sanity check” in EEG-fMRI.

## Electroencephalography-functional magnetic resonance imaging analysis methods

**FIGURE 11:**
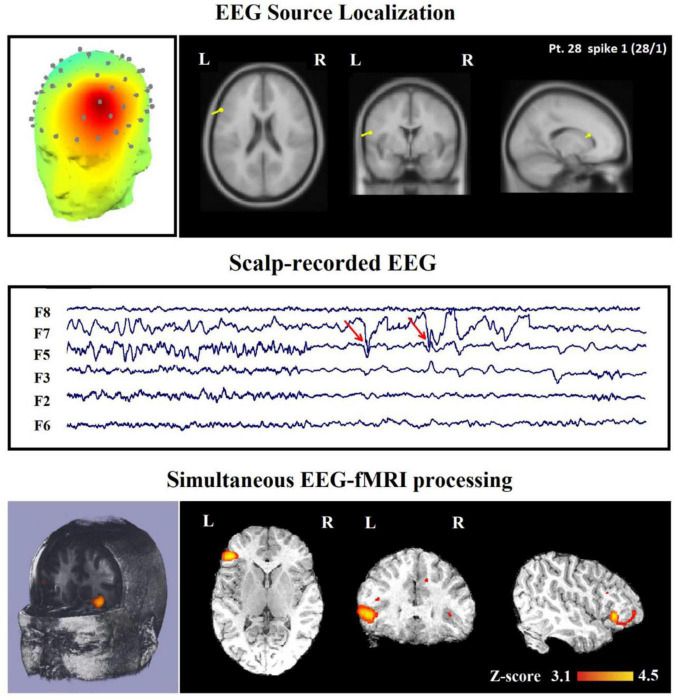
Source analysis using EEG-informed fMRI approach in a 26-year-old woman with frontal lobe epilepsy (FLE) which demonstrates a neocortical activation in the inferior frontal gyrus. Top, the component identified on scalp EEG is shown which is located in the left frontotemporal lobe (left) and the dipole localization of the identified generator in deep brain structures (right). Middle, scalp recorded EEG with marked events on F3, F5, and F7. Bottom, Localization of the generator applying simultaneous analysis of EEG-fMRI. The active area is marked with a yellow-red color (Ebrahimzadeh et al., 2021b).

For simultaneous multimodal acquisitions and especially EEG-fMRI, the way to analyze the data plays a significant role in order to get valuable results. Two macro-areas can group the variety of analyses used in the literature: symmetric analyses and integrated analyses (Ahmad et al., 2016). Overall, the symmetrical approach is concerned with analyzing the outcome of two modalities at the same time, whereas the integrated analysis uses the data obtained from one of the modalities to validate the data acquired from the other one. Accordingly, generating a unique model to facilitate the investigation of the brain activity is not impossible (Huster et al., 2012). The integrated analysis can be done in one of these two practical ways: fMRI-informed EEG analysis (Lei et al., 2015) and EEG-informed fMRI analysis (Andreou et al., 2017). In the first one, the activation areas on fMRI data are used as a base to analyze and correct the EEG sources (Babiloni et al., 2002), while in the second one, the electrical activities and EEG parameters are used to predict the hemodynamic variations in the fMRI data (Figure 11; Abreu et al., 2018). Nevertheless, there is still an open issue for choosing the optimal way to analyze the simultaneous EEG-fMRI data. Revealing actually meaningful quantitative biomarkers to use for characterizing the physiological and pathological activity of the brain requires further investigation.

## Discussion and future perspectives

Using a combination of EEG and functional magnetic resonance imaging (fMRI), researchers can gain a better understanding of the neural basis of behavior, including brain function and malfunction. The purpose of this work is to examine current knowledge and research on the utilization of fMRI and EEG data in combination. We looked at the complementing qualities of the two methodologies briefly before going over how to get the two forms of data. We investigated the respective benefits and drawbacks of doing so simultaneously, and strategies and methods for efficiently combining them. Topics related to clinical applications were discussed.

**FIGURE 12:**
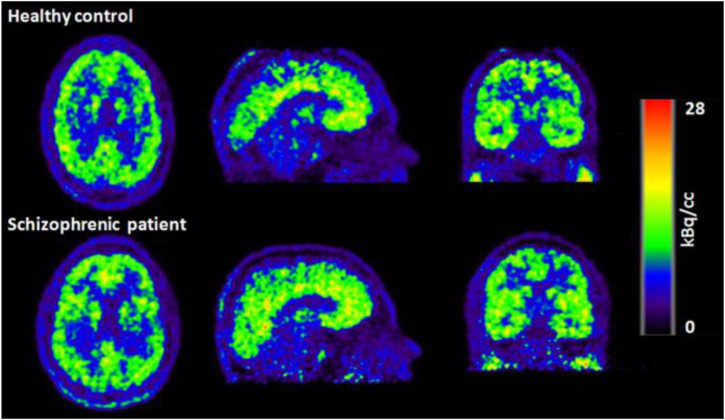
PET images of the healthy control (top row) and schizophrenic patient (bottom row) acquired during the first resting state measurement (Neuner et al., 2019).

**FIGURE 13:**
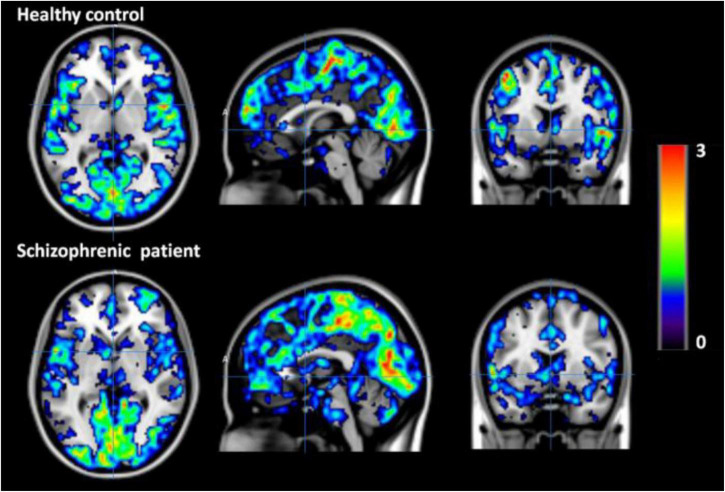
fMRI Degree Centrality (DC) values of healthy control (top row) and schizophrenic patient (bottom row) during first resting state measurement. The DC values are overlaid on the MNI template. DC is one of the data driven functional connectivity measures and is computed using rs-fMRI data from both the healthy control and the schizophrenic patient (Neuner et al., 2019).

Simultaneous EEG-fMRI is a valuable non-invasive approach to study the human brain function. This paper explores most recent EEG-fMRI studies focused on advanced analyses for mapping the brain networks. These two modalities have been renowned for being complement due to the good temporal resolution of EEG and the good spatial resolution of fMRI. However, the characterization of molecular processes that subtend resting state analysis or a specific task cannot be achieved through these tools. For this aim, a trimodal approach has been proposed which is integrating an MR-compatible EEG system in a hybrid MR–PET scanner (Shah et al., 2013). This can provide a broader view of the brain activity although the PET attenuation by the EEG-hat can derive technical issues (Rajkumar et al., 2017; Neuner et al., 2019; Figures 12, 13).

The clinical interest to provide a better representation and localization of neural activity, combining the hemodynamic changes in association with the epileptic discharges on brain’s surface was the initial motivation to evolve simultaneous EEG-fMRI recordings (Ives et al., 1993). To date, the simultaneous EEG-fMRI fusion offers several intriguing clinical applications, providing vital information and insights into how the human brain operates. The goal of this work is to give a brief overview of what is currently known about simultaneous EEG-fMRI data fusion, and to provide a (maturity) metric system for evaluating and comparing the (multivariate) methodologies and techniques used to fuse and analyze data derived from simultaneous EEG and fMRI recording.

## Conclusion

Simultaneous recording of EEG and fMRI enables a reference tool to investigate physiological brain networks and study the correspondence between the brain hemodynamic signal and electrical neural activity. More importantly, it can help to characterize the correlation of regional brain activity with EEG spectrum and provide new insights and hopefully treatment targets on psychiatric and neurological diseases. Multi-modal neuroimaging and especially simultaneous EEG-fMRI are becoming widespread in the literature of brain science. However, the optimal integrated and standardized analysis is still a challenge that lies ahead with technological development. A considerable progress on this issue has been made during recent years in order to achieve the optimal data acquisition, study design, and data analysis. Significant improvements in MRI data analysis and artifact removal methods can lead fully continuous EEG-fMRI recordings to become the mainstay of multi-modal functional neuroimaging in the future, but to achieve this, the algorithms and procedures of EEG and fMRI biophysical modeling will need further developments and research to provide a better integration between the temporal information of EEG and spatial information of fMRI data. Artifact reduction procedures are also necessary to be validated using computer stimulations as they have been done in a small number of studies. We believe that the clinical applications of simultaneous EEG-fMRI can definitely go beyond the epilepsy research and provide new insights into the dynamical bases of neurological, psychiatric, and neurodevelopmental disorders.

## Author contributions

EE contributed substantially to the conception and design of the work. EE, SS, SMS, MS, HO, and FF prepared the literature data base and drafted the work. EE, FF, LR, and HS-Z revised critically the manuscript for important intellectual content. HS-Z provided approval for publication of the content. All authors read and approved the final manuscript.

## References

[B1] Abreu R. Leal A. Figueiredo P. (2018). EEG-informed fMRI: a review of data analysis methods. *Front. Hum. Neurosci.* 12:29. 10.3389/fnhum.2018.00029 29467634 PMC5808233

[B2] Acharjee P. P. Phlypo R. Wu L. Calhoun V. D. Adalı T. (2015). Independent vector analysis for gradient artifact removal in concurrent EEG-fMRI data. *IEEE Trans. Biomed. Eng.* 62 1750–1758. 10.1109/TBME.2015.2403298 25700437

[B3] Aghakhani Y. Bagshaw A. P. Bénar C. G. Hawco C. Andermann F. Dubeau F. (2004). fMRI activation during spike and wave discharges in idiopathic generalized epilepsy. *Brain* 127 1127–1144. 10.1093/brain/awh136 15033899

[B4] Ahmad R. F. Malik A. S. Kamel N. Reza F. Abdullah J. M. (2016). Simultaneous EEG-fMRI for working memory of the human brain. *Australas. Phys. Eng. Sci. Med.* 39 363–378. 10.1007/S13246-016-0438-X 27043850

[B5] Ahmad R. F. Malik A. S. Kamel N. Reza F. Karim A. H. A. (2015). “Optimization and development of concurrent EEG-fMRI data acquisition setup for understanding neural mechanisms of brain,” in *Proceedings of the 2015 IEEE International Instrumentation and Measurement Technology Conference (I2MTC)*, (Pisa: IEEE), 476–481. 10.1109/I2MTC.2015.7151314

[B6] Aiello M. Cavaliere C. Salvatore M. (2016). Hybrid PET/MR imaging and brain connectivity. *Front. Neurosci.* 10:64. 10.3389/FNINS.2016.00064 26973446 PMC4771762

[B7] Aiello M. Cavaliere C. Marchitelli R. d’Albore A. De Vita E. Salvatore M. (2018). Hybrid PET/MRI methodology. *Int. Rev. Neurobiol.* 141 97–128. 10.1016/BS.IRN.2018.07.026 30314608

[B8] Aiello M. Salvatore E. Cachia A. Pappatà S. Cavaliere C. Prinster A. (2015). Relationship between simultaneously acquired resting-state regional cerebral glucose metabolism and functional MRI: a PET/MR hybrid scanner study. *Neuroimage* 113 111–121. 10.1016/J.NEUROIMAGE.2015.03.017 25791784

[B9] Aminoff M. J. (2005). “Chapter 3 – Electroencephalography: general principles and clinical applications,” in *Electrodiagnosis in Clinical Neurology, 5th Edn*. eds Aminoff M. J. (London: Churchill Livingstone), 37–84. 10.1016/B978-0-443-06647-4.50008-2

[B10] Amoozegar S. Pooyan M. Ebrahimzadeh E. (2013). Classification of brain signals in normal subjects and patients with epilepsy using mixture of experts. *Comput. Intell. Electr. Eng.* 4 1–8.

[B11] An D. Dubeau F. Gotman J. (2015). BOLD responses related to focal spikes and widespread bilateral synchronous discharges generated in the frontal lobe. *Epilepsia* 56 366–374. 10.1111/epi.12909 25599979

[B12] Andreou C. Frielinghaus H. Rauh J. Mußmann M. Vauth S. Braun P. (2017). Theta and high-beta networks for feedback processing: a simultaneous EEG-fMRI study in healthy male subjects. *Transl. Psychiatry* 7:e1016. 10.1038/tp.2016.287 28140398 PMC5299393

[B13] Astolfi L. Cincotti F. Mattia D. Marciani M. G. Baccala L. A. Fallani F. D. V. (2007). Comparison of different cortical connectivity estimators for high-resolution EEG recordings. *Hum. Brain Mapp.* 28 143–157. 10.1002/HBM.20263 16761264 PMC6871398

[B14] Auletta L. Gramanzini M. Gargiulo S. Albanese S. Salvatore M. Greco A. (2017). Advances in multimodal molecular imaging. *Q. J. Nucl. Med. Mol. Imaging* 61 19–32. 10.23736/S1824-4785.16.02943-5 27858404

[B15] Babiloni F. Babiloni C. Carducci F. Del Gratta C. Romani G. L. Rossini P. M. (2002). Cortical source estimate of combined high resolution EEG and fMRI data related to voluntary movements. *Methods Inf. Med.* 41 443–450. 10.1055/S-0038-1634217 12501818

[B16] Bagshaw A. P. Kobayashi E. Dubeau F. Pike G. B. Gotman J. (2006). Correspondence between EEG-fMRI and EEG dipole localisation of interictal discharges in focal epilepsy. *Neuroimage* 30 417–425. 10.1016/j.neuroimage.2005.09.033 16269248

[B17] Ball T. Schreiber A. Feige B. Wagner M. Lücking C. H. Kristeva-Feige R. (1999). The role of higher-order motor areas in voluntary movement as revealed by high-resolution EEG and fMRI. *Neuroimage* 10 682–694. 10.1006/nimg.1999.0507 10600414

[B18] Beers C. A. Williams R. J. Gaxiola-Valdez I. Pittman D. J. Kang A. T. Aghakhani Y. (2015). Patient specific hemodynamic response functions associated with interictal discharges recorded via simultaneous intracranial EEG-fMRI. *Hum. Brain Mapp.* 36 5252–5264. 10.1002/hbm.23008 26417648 PMC6869833

[B19] Beres A. M. (2017). Time is of the essence: a review of electroencephalography (EEG) and event-related brain potentials (ERPs) in language research. *Appl. Psychophysiol. Biofeedback* 42 247–255. 10.1007/S10484-017-9371-3 28698970 PMC5693972

[B20] Bonmassar G. Anami K. Ives J. Belliveau J. W. (1999). Visual evoked potential (VEP) measured by simultaneous 64-channel EEG and 3T fMRI. *Neuroreport* 10 1893–1897. 10.1097/00001756-199906230-00018 10501528

[B21] Bonmassar G. Schwartz D. P. Liu A. K. Kwong K. K. Dale A. M. Belliveau J. W. (2001b). Spatiotemporal brain imaging of visual-evoked activity using interleaved EEG and fMRI recordings. *Neuroimage* 13 1035–1043. 10.1006/nimg.2001.0754 11352609

[B22] Bonmassar G. Hadjikhani N. Ives J. R. Hinton D. Belliveau J. W. (2001a). Influence of EEG electrodes on the BOLD fMRI signal. *Hum. Brain Mapp.* 14 108–115. 10.1002/hbm.1045 11500994 PMC6871948

[B23] Bonmassar G. Purdon P. L. Jääskeläinen I. P. Chiappa K. Solo V. Brown E. N. (2002). Motion and ballistocardiogram artifact removal for interleaved recording of EEG and EPs during MRI. *Neuroimage* 16 1127–1141. 10.1006/nimg.2002.1125 12202099

[B24] Bridwell D. Calhoun V. (2019). “Fusing Concurrent EEG and fMRI intrinsic networks,” in *Magnetoencephalography*, eds Supek S. Aine C. (Cham: Springer), 293–315.

[B25] Briels C. T. Briels C. T. Schoonhoven D. N. Schoonhoven D. N. Stam C. J. De Waal H. (2020). Reproducibility of EEG functional connectivity in Alzheimer’s disease. *Alzheimers Res. Ther.* 12 1–14. 10.1186/S13195-020-00632-3/TABLES/3 PMC7271479 32493476

[B26] Britz J. Van De Ville D. Michel C. M. (2010). BOLD correlates of EEG topography reveal rapid resting-state network dynamics. *Neuroimage* 52 1162–1170. 10.1016/j.neuroimage.2010.02.052 20188188

[B27] Brueggen K. Fiala C. Berger C. Ochmann S. Babiloni C. Teipel S. J. (2017). Early changes in alpha band power and DMN BOLD activity in Alzheimer’s disease: a simultaneous resting state EEG-fMRI study. *Front. Aging Neurosci.* 9:319. 10.3389/fnagi.2017.00319 29056904 PMC5635054

[B28] Bruno M. A. Fernández-Espejo D. Lehembre R. Tshibanda L. Vanhaudenhuyse A. Gosseries O. (2011). Multimodal neuroimaging in patients with disorders of consciousness showing “functional hemispherectomy.”. *Prog. Brain Res.* 193 323–333. 10.1016/B978-0-444-53839-0.00021-1 21854972

[B29] Buxton R. B. (2002). *Introduction to Functional Magnetic Resonance Imaging.* Cambridge: Cambridge University Press. 10.1017/CBO9780511549854

[B30] Buzsáki G. Watson B. O. (2022). Brain rhythms and neural syntax: implications for efficient coding of cognitive content and neuropsychiatric disease. *Dialogues Clin. Neurosci.* 14 10.31887/DCNS.2012.14.4/gbuzsaki 23393413 PMC3553572

[B31] Carlen M. Meletis K. Siegle J. H. Cardin J. A. Futai K. Vierling-Claassen D. (2012). A critical role for NMDA receptors in parvalbumin interneurons for gamma rhythm induction and behavior. *Mol. Psychiatry* 17 537–548. 10.1038/mp.2011.31 21468034 PMC3335079

[B32] Cavaliere C. Kandeepan S. Aiello M. De Paula D. R. Marchitelli R. Fiorenza S. (2018a). Multimodal neuroimaging approach to variability of functional connectivity in disorders of consciousness: a PET/MRI pilot study. *Front. Neurol.* 9:861. 10.3389/FNEUR.2018.00861 30405513 PMC6200912

[B33] Cavaliere C. Longarzo M. Orsini M. Aiello M. Grossi D. (2018b). Fronto-temporal circuits in musical hallucinations: a PET-MR case study. *Front. Hum. Neurosci.* 12:385. 10.3389/FNHUM.2018.00385 30319380 PMC6170624

[B34] Chang C. Liu Z. Chen M. C. Liu X. Duyn J. H. (2013). EEG correlates of time-varying BOLD functional connectivity. *Neuroimage* 72 227–236. 10.1016/j.neuroimage.2013.01.049 23376790 PMC3602157

[B35] Christmann C. Ruf M. Braus D. F. Flor H. (2002). Simultaneous electroencephalography and functional magnetic resonance imaging of primary and secondary somatosensory cortex in humans after electrical stimulation. *Neurosci. Lett.* 333 69–73. 10.1016/S0304-3940(02)00969-2 12401562

[B36] Colgin L. L. (2011). Oscillations and hippocampal–prefrontal synchrony. *Curr. Opin. Neurobiol.* 21 467–474. 10.1016/j.conb.2011.04.006 21571522 PMC3578407

[B37] Czisch M. Wehrle R. Kaufmann C. Wetter T. C. Holsboer F. Pollmächer T. (2004). Functional MRI during sleep: BOLD signal decreases and their electrophysiological correlates. *Eur. J. Neurosci.* 20 566–574. 10.1111/j.1460-9568.2004.03518.x 15233766

[B38] Debener S. Ullsperger M. Siegel M. Engel A. K. (2006). Single-trial EEG-fMRI reveals the dynamics of cognitive function. *Trends Cogn. Sci.* 10 558–563. 10.1016/J.TICS.2006.09.010 17074530

[B39] Deligianni F. Centeno M. Carmichael D. W. Clayden J. D. (2014). Relating resting-state fMRI and EEG whole-brain connectomes across frequency bands. *Front. Neurosci.* 8:258. 10.3389/fnins.2014.00258 25221467 PMC4148011

[B40] Detre J. A. (2004). fMRI: applications in epilepsy. *Epilepsia* 45 26–31. 10.1111/j.0013-9580.2004.04006.x 15281954

[B41] Dong L. Luo C. Zhu Y. Hou C. Jiang S. Wang P. (2016). Complex discharge-affecting networks in juvenile myoclonic epilepsy: a simultaneous EEG-fMRI study. *Hum. Brain Mapp.* 37 3515–3529. 10.1002/hbm.23256 27159669 PMC6867253

[B42] Ebrahimzadeh E. Shams M. Seraji M. Sadjadi S. M. Rajabion L. Soltanian-Zadeh H. (2021b). Localizing epileptic foci using simultaneous EEG-fMRI recording: template component cross-correlation. *Front. Neurol.* 12:695997. 10.3389/fneur.2021.695997 34867704 PMC8634837

[B43] Ebrahimzadeh E. Alavi S. M. Bijar A. Pakkhesal A. (2013). A novel approach for detection of deception using smoothed pseudo Wigner-Ville distribution (SPWVD). *J. Biomed. Sci. Eng.* 6 8–18. 10.4236/jbise.2013.61002

[B44] Ebrahimzadeh E. Soltanian-Zadeh H. Nadjar Araabi B. (2018b). Localization of epileptic focus using simultaneously acquired EEG-FMRI data. *Comput. Intell. Electr. Eng.* 9 15–28. 10.22108/isee.2018.111024.1123

[B45] Ebrahimzadeh E. Fayaz F. Nikravan M. Ahmadi F. Dolatabad M. R. (2018a). Towards an automatic diagnosis system for lumbar disc herniation: the significance of local subset feature selection. *Biomed. Eng. Appl. Basis Commun.* 30:1850044. 10.4015/S1016237218500448

[B46] Ebrahimzadeh E. Soltanian-Zadeh H. Araabi B. N. Fesharaki S. S. H. Habibabadi J. M. (2019b). Component-related BOLD response to localize epileptic focus using simultaneous EEG-fMRI recordings at 3T. *J. Neurosci. Methods* 322 34–49. 10.1016/j.jneumeth.2019.04.010 31026487

[B47] Ebrahimzadeh E. Shams M. Fayaz F. Rajabion L. Mirbagheri M. Nadjar Araabi B. (2019a). Quantitative determination of concordance in localizing epileptic focus by component-based EEG-fMRI. *Comput. Methods Programs Biomed.* 177 231–241. 10.1016/j.cmpb.2019.06.003 31319952

[B48] Ebrahimzadeh E. Shams M. Rahimpour Jounghani A. Fayaz F. Mirbagheri M. Hakimi N. (2021a). Localizing confined epileptic foci in patients with an unclear focus or presumed multifocality using a component-based EEG-fMRI method. *Cogn. Neurodyn.* 15 207–222. 10.1007/s11571-020-09614-5 33854640 PMC7969677

[B49] Ebrahimzadeh E. Soltanian-Zadeh H. Araabi B. N. Fesharaki S. S. H. Habibabadi J. M. Nadjar Araabi B. (2019). Localizing epileptic focus through simultaneous EEG-fMRI recording and automated detection of IED from inside-scanner EEG. *Iran J. Biomed. Eng.* 13 135–145. 10.22041/IJBME.2019.103479.1447

[B50] Federico P. Archer J. S. Abbott D. F. Jackson G. D. (2005). Cortical/subcortical BOLD changes associated with epileptic discharges an EEG-fMRI study at 3 T. *Neurology* 64 1125–1130. 10.1212/01.WNL.0000156358.72670.AD 15824333

[B51] Figley C. R. Stroman P. W. (2011). The role(s) of astrocytes and astrocyte activity in neurometabolism, neurovascular coupling, and the production of functional neuroimaging signals. *Eur. J. Neurosci.* 33 577–588. 10.1111/J.1460-9568.2010.07584.X 21314846

[B52] Fisch L. Privman E. Ramot M. Harel M. Nir Y. Kipervasser S. (2009). Neural “ignition”: enhanced activation linked to perceptual awareness in human ventral stream visual cortex. *Neuron* 64 562–574. 10.1016/j.neuron.2009.11.001 19945397 PMC2854160

[B53] Fleury M. Barillot C. Mano M. Bannier E. Maurel P. (2019). Automated electrodes detection during simultaneous EEG/fMRI. *Front. ICT* 5:31. 10.3389/fict.2018.00031

[B54] Franciotti R. Falasca N. W. Bonanni L. Anzellotti F. Maruotti V. Comani S. (2013). Default network is not hypoactive in dementia with fluctuating cognition: an Alzheimer disease/dementia with Lewy bodies comparison. *Neurobiol. Aging* 34 1148–1158. 10.1016/j.neurobiolaging.2012.09.015 23063646

[B55] Fujisawa S. Buzsáki G. (2011). A 4 Hz oscillation adaptively synchronizes prefrontal, VTA, and hippocampal activities. *Neuron* 72 153–165. 10.1016/j.neuron.2011.08.018 21982376 PMC3235795

[B56] George J. S. Aine C. J. Mosher J. C. Schmidt D. M. Ranken D. M. Schlitt H. A. (1995). Mapping function in the human brain with magnetoencephalography, anatomical magnetic resonance imaging, and functional magnetic resonance imaging. *J. Clin. Neurophysiol. Off. Publ. Am. Electroencephalogr. Soc.* 12 406–431. 10.1097/00004691-199509010-00002 8576388

[B57] Gholipour T. You X. Stufflebeam S. M. Loew M. Koubeissi M. Z. Morgan V. L. (2022). Common functional connectivity alterations in focal epilepsies identified by machine learning. *Epilepsia* 63 629–640. 10.1111/EPI.17160 34984672 PMC9022014

[B58] Goldman R. I. Stern J. M. Engel J. Jr. (2002). Cohen MS. Simultaneous EEG and fMRI of the alpha rhythm. *Neuroreport* 13:2487. 10.1097/00001756-200212200-00022 12499854 PMC3351136

[B59] Grassi R. Cavaliere C. Cozzolino S. Mansi L. Cirillo S. Tedeschi G. (2009). Small animal imaging facility: new perspectives for the radiologist. *Radiol. Med.* 114 152–167. 10.1007/s11547-008-0352-8 19082783

[B60] Greco A. Ragucci M. Liuzzi R. Gargiulo S. Gramanzini M. Coda A. R. D. (2013). Repeatability, reproducibility and standardisation of a laser Doppler imaging technique for the evaluation of normal mouse hindlimb perfusion. *Sensors (Switzerland)* 13 500–515. 10.3390/S130100500 PMC3574687 23275085

[B61] Guo Q. Zhou T. Li W. Dong L. Wang S. Zou L. (2017). Single-trial EEG-informed fMRI analysis of emotional decision problems in hot executive function. *Brain Behav.* 7:e00728. 10.1002/BRB3.728 28729935 PMC5516603

[B62] Hall D. A. Haggard M. P. Akeroyd M. A. Palmer A. R. Summerfield A. Q. Elliott M. R. (1999). “Sparse” temporal sampling in auditory fMRI. *Hum. Brain Mapp.* 7 213–223. 10.1002/(SICI)1097-0193(1999)7:3<213::AID-HBM5>3.0.CO;2-N 10194620 PMC6873323

[B63] Heinze H. J. Mangun G. R. Burchert W. Hinrichs H. Scholz M. Münte T. F. (1994). Combined spatial and temporal imaging of brain activity during visual selective attention in humans. *Nature* 372 543–546. 7990926 10.1038/372543a0

[B64] Herrmann C. S. Debener S. (2008). Simultaneous recording of EEG and BOLD responses: a historical perspective. *Int. J. Psychophysiol.* 67 161–168. 10.1016/j.ijpsycho.2007.06.006 17719112

[B65] Herrmann C. S. Fründ I. Lenz D. (2010). Human gamma-band activity: a review on cognitive and behavioral correlates and network models. *Neurosci. Biobehav. Rev.* 34 981–992. 10.1016/j.neubiorev.2009.09.001 19744515

[B66] Herweg N. A. Apitz T. Leicht G. Mulert C. Fuentemilla L. Bunzeck N. (2016). Theta-alpha oscillations bind the hippocampus, prefrontal cortex, and striatum during recollection: evidence from simultaneous EEG–fMRI. *J. Neurosci.* 36 3579–3587. 10.1523/JNEUROSCI.3629-15.2016 27013686 PMC6601728

[B67] Hlinka J. Alexakis C. Diukova A. Liddle P. F. Auer D. P. (2010). Slow EEG pattern predicts reduced intrinsic functional connectivity in the default mode network: an inter-subject analysis. *Neuroimage* 53 239–246. 10.1016/j.neuroimage.2010.06.002 20538065

[B68] Huang Y. Koestner M. L. Ackers G. K. (1996). Heterotropic effects of chloride on the ligation microstates of hemoglobin at constant water activity. *Biophys. J.* 71 2106–2116. 10.1016/S0006-3495(96)79409-2 8889185 PMC1233677

[B69] Huster R. J. Debener S. Eichele T. Herrmann C. S. (2012). Methods for simultaneous EEG-fMRI: an introductory review. *J. Neurosci.* 32 6053–6060. 10.1523/JNEUROSCI.0447-12.2012 22553012 PMC6622140

[B70] Iannaccone R. Hauser T. U. Staempfli P. Walitza S. Brandeis D. Brem S. (2015). Conflict monitoring and error processing: new insights from simultaneous EEG-fMRI. *Neuroimage* 105 395–407. 10.1016/J.NEUROIMAGE.2014.10.028 25462691

[B71] Ives J. R. Warach S. Schmitt F. Edelman R. R. Schomer D. L. (1993). Monitoring the patient’s EEG during echo planar MRI. *Electroencephalogr. Clin. Neurophysiol.* 87 417–420. 10.1016/0013-4694(93)90156-P 7508375

[B72] Jackson A. F. Bolger D. J. (2014). The neurophysiological bases of EEG and EEG measurement: a review for the rest of us. *Psychophysiology* 51 1061–1071. 10.1111/PSYP.12283 25039563

[B73] Javitt D. C. (2012). “Glycine transport inhibitors in the treatment of schizophrenia,” in *Novel Antischizophrenia Treatments. Handbook of Experimental Pharmacology*, Vol. 213, eds Geyer M. Gross G. (Berlin: Springer). 10.1007/978-3-642-25758-2_1 23027421

[B74] Jueptner M. Weiller C. (1995). Does measurement of regional cerebral blood flow reflect synaptic activity?—Implications for PET and fMRI. *Neuroimage* 2 148–156. 10.1006/NIMG.1995.1017 9343597

[B75] Keinänen T. Rytky S. Korhonen V. Huotari N. Nikkinen J. Tervonen O. (2018). Fluctuations of the EEG-fMRI correlation reflect intrinsic strength of functional connectivity in default mode network. *J. Neurosci. Res.* 96 1689–1698. 10.1002/jnr.24257 29761531

[B76] Klem G. H. Lüders H. O. Jasper H. H. Elger C. (1961). The ten twenty electrode system: international federation of societies for electroencephalography and clinical neurophysiology. *Am. J. EEG Technol.* 1 13–19. 10.1080/00029238.1961.11080571 10590970

[B77] Klimesch W. Sauseng P. Hanslmayr S. (2007). EEG alpha oscillations: the inhibition–timing hypothesis. *Brain Res. Rev.* 53 63–88. 10.1016/j.brainresrev.2006.06.003 16887192

[B78] Knaut P. von Wegner F. Morzelewski A. Laufs H. (2019). EEG-correlated fMRI of human alpha (de-)synchronization. *Clin. Neurophysiol.* 130 1375–1386. 10.1016/J.CLINPH.2019.04.715 31220698

[B79] Krakow K. Woermann F. G. Symms M. R. Allen P. J. Lemieux L. Barker G. J. (1999). EEG-triggered functional MRI of interictal epileptiform activity in patients with partial seizures. *Brain* 122(Pt 9) 1679–1688. 10.1093/brain/122.9.1679 10468507

[B80] Kristeva-Feige R. Walter H. Lütkenhöner B. Hampson S. Ross B. Knorr U. (1994). A neuromagnetic study of the functional organization of the sensorimotor cortex. *Eur. J. Neurosci.* 6 632–639. 10.1111/j.1460-9568.1994.tb00308.x 8025715

[B81] Kugel H. Bremer C. Püschel M. Fischbach R. Lenzen H. Tombach B. (2003). Hazardous situation in the MR bore: induction in ECG leads causes fire. *Eur. Radiol.* 13 690–694. 10.1007/S00330-003-1841-8 12664104

[B82] Laufs H. Kleinschmidt A. Beyerle A. Eger E. Salek-Haddadi A. Preibisch C. (2003a). EEG-correlated fMRI of human alpha activity. *Neuroimage* 19 1463–1476. 10.1016/S1053-8119(03)00286-6 12948703

[B83] Laufs H. Krakow K. Sterzer P. Eger E. Beyerle A. Salek-Haddadi A. (2003b). Electroencephalographic signatures of attentional and cognitive default modes in spontaneous brain activity fluctuations at rest. *Proc. Natl. Acad. Sci. U.S.A.* 100 11053–11058. 10.1073/pnas.1831638100 12958209 PMC196925

[B84] Laufs H. Lengler U. Hamandi K. Kleinschmidt A. Krakow K. (2006). Linking generalized spike-and-wave discharges and resting state brain activity by using EEG/fMRI in a patient with absence seizures. *Epilepsia* 47 444–448. 10.1111/j.1528-1167.2006.00443.x 16499775

[B85] Lazeyras F. Zimine I. Blanke O. Perrig S. H. Seeck M. (2001). Functional MRI with simultaneous EEG recording: feasibility and application to motor and visual activation. *J. Magn. Reson. Imaging Off. J. Int. Soc. Magn. Reson. Med.* 13 943–948. 10.1002/jmri.1135 11382957

[B86] Lehmann D. Faber P. L. Galderisi S. Herrmann W. M. Kinoshita T. Koukkou M. (2005). EEG microstate duration and syntax in acute, medication-naive, first-episode schizophrenia: a multi-center study. *Psychiatry Res. Neuroimaging* 138 141–156. 10.1016/j.pscychresns.2004.05.007 15766637

[B87] Lehmann D. Ozaki H. Pál I. (1987). EEG alpha map series: brain micro-states by space-oriented adaptive segmentation. *Electroencephalogr. Clin. Neurophysiol.* 67 271–288. 10.1016/0013-4694(87)90025-3 2441961

[B88] Lei X. Wu T. Valdes-Sosa P. A. (2015). Incorporating priors for EEG source imaging and connectivity analysis. *Front. Neurosci.* 9:284. 10.3389/FNINS.2015.00284 26347599 PMC4539512

[B89] Leicht G. Kirsch V. Giegling I. Karch S. Hantschk I. Möller H.-J. (2010). Reduced early auditory evoked gamma-band response in patients with schizophrenia. *Biol. Psychiatry* 67 224–231. 10.1016/j.biopsych.2009.07.033 19765689

[B90] Lemieux L. (2004). Electroencephalography-correlated functional MR imaging studies of epileptic activity. *Neuroimaging Clin.* 14 487–506. 10.1016/j.nic.2004.04.009 15324860

[B91] Lemieux L. Salek-haddadi A. Josephs O. Allen P. Toms N. Scott C. (2001). Event-related fMRI with simultaneous and continuous EEG?: description of the method and initial case report. *Neuroimage* 14 780–787. 10.1006/nimg.2001.0853 11506550

[B92] Liebenthal E. Ellingson M. L. Spanaki M. V. Prieto T. E. Ropella K. M. Binder J. R. (2003). Simultaneous ERP and fMRI of the auditory cortex in a passive oddball paradigm. *Neuroimage* 19 1395–1404. 10.1016/s1053-8119(03)00228-3 12948697

[B93] Liston A. D. De Munck J. C. Hamandi K. Laufs H. Ossenblok P. Duncan J. S. (2006). Analysis of EEG-fMRI data in focal epilepsy based on automated spike classification and signal space projection. *Neuroimage* 31 1015–1024. 10.1016/j.neuroimage.2006.01.040 16545967

[B94] Liu Y. Wang K. Yu C. He Y. Zhou Y. Liang M. (2008). Regional homogeneity, functional connectivity and imaging markers of Alzheimer’s disease: a review of resting-state fMRI studies. *Neuropsychologia* 46 1648–1656. 10.1016/J.NEUROPSYCHOLOGIA.2008.01.027 18346763

[B95] Makeig S. Westerfield M. Jung T.-P. Enghoff S. Townsend J. Courchesne E. (2002). Dynamic brain sources of visual evoked responses. *Science* 295 690–694. 10.1126/science.1066168 11809976

[B96] Manganas S. Bourbakis N. (2017). “A comparative survey on simultaneous EEG-fMRI methodologies,” in *Proceedings of the 2017 IEEE 17th International Conference on Bioinformatics and Bioengineering (BIBE)*, (Washington, DC: IEEE), 1–8. 10.1093/sleep/zsy056

[B97] Marawar R. A. Yeh H. J. Carnabatu C. J. Stern J. M. (2017). Functional MRI correlates of resting-state temporal theta and delta EEG rhythms. *J. Clin. Neurophysiol.* 34 69–76. 10.1097/WNP.0000000000000309 27763967

[B98] Marchitelli R. Aiello M. Cachia A. Quarantelli M. Cavaliere C. Postiglione A. (2018). Simultaneous resting-state FDG-PET/fMRI in Alzheimer disease: relationship between glucose metabolism and intrinsic activity. *Neuroimage* 176 246–258. 10.1016/j.neuroimage.2018.04.048 29709628

[B99] Marchitelli R. Minati L. Marizzoni M. Bosch B. Bartrés-Faz D. Müller B. W. (2016). Test-retest reliability of the default mode network in a multi-centric fMRI study of healthy elderly: effects of data-driven physiological noise correction techniques. *Hum. Brain Mapp.* 37 2114–2132. 10.1002/HBM.23157 26990928 PMC6867386

[B100] Marino M. Liu Q. Koudelka V. Porcaro C. Hlinka J. Wenderoth N. (2018). Adaptive optimal basis set for BCG artifact removal in simultaneous EEG-fMRI. *Sci. Rep.* 8:8902. 10.1038/S41598-018-27187-6 29891929 PMC5995808

[B101] Mele G. Cavaliere C. Alfano V. Orsini M. Salvatore M. Aiello M. (2019). Simultaneous EEG-fMRI for functional neurological assessment. *Front. Neurol.* 10:848. 10.3389/fneur.2019.00848 31456735 PMC6700249

[B102] Menon R. S. Kim S. G. (1999). Spatial and temporal limits in cognitive neuroimaging with fMRI. *Trends Cogn. Sci.* 3 207–216. 10.1016/S1364-6613(99)01329-7 10354573

[B103] Menon V. Ford J. M. Lim K. O. Glover G. H. Pfefferbaum A. (1997). Combined event-related fMRI and EEG evidence for temporal—parietal cortex activation during target detection. *Neuroreport* 8 3029–3037. 10.1097/00001756-199709290-00007 9331910

[B104] Metwali H. Raemaekers M. Kniese K. Kardavani B. Fahlbusch R. Samii A. (2019). Reliability of functional magnetic resonance imaging in patients with brain tumors: a critical review and meta-analysis. *World Neurosurg.* 125 183–190. 10.1016/J.WNEU.2019.01.194 30743033

[B105] Mirbagheri M. Hakimi N. Ebrahimzadeh E. Setarehdan S. K. K. (2020a). Quality analysis of heart rate derived from functional near-infrared spectroscopy in stress assessment. *Inform. Med. Unlocked* 18:100286. 10.1016/j.imu.2019.100286

[B106] Mirbagheri M. Hakimi N. Ebrahimzadeh E. Setarehdan S. K. (2020b). Simulation and in vivo investigation of light-emitting diode, near infrared Gaussian beam profiles. *J. Near Infrared Spectrosc.* 28 37–50. 10.1177/0967033519884209

[B107] Mirbagheri M. Hakimi N. Ebrahimzadeh E. Pourrezaei K. Kamaledin Setarehdan S. Setarehdan S. K. (2019). Enhancement of optical penetration depth of LED-based NIRS systems by comparing different beam profiles. *Biomed. Phys. Eng. Express.* 5:65004. 10.1088/2057-1976/ab42d9

[B108] Moeller F. Siniatchkin M. Gotman J. (2020). “Simultaneous EEG and fMRI recordings (EEG–fMRI),” in *fMRI*, eds Ulmer S. Jansen O. (Cham: Springer), 175–191.

[B109] Moosmann M. Ritter P. Krastel I. Brink A. Thees S. Blankenburg F. (2003). Correlates of alpha rhythm in functional magnetic resonance imaging and near infrared spectroscopy. *Neuroimage* 20 145–158. 10.1016/s1053-8119(03)00344-6 14527577

[B110] Mulert C. (2022). Simultaneous EEG and fMRI: towards the characterization of structure and dynamics of brain networks. *Dialogues Clin. Neurosci.* 15 10.31887/DCNS.2013.15.3/cmulert 24174908 PMC3811108

[B111] Mulert C. Lemieux L. (2009). *EEG-fMRI: Physiological Basis, Technique, and Applications.* Berlin: Springer Science & Business Media.

[B112] Mulert C. Jäger L. Schmitt R. Bussfeld P. Pogarell O. Möller H.-J. (2004). Integration of fMRI and simultaneous EEG: towards a comprehensive understanding of localization and time-course of brain activity in target detection. *Neuroimage* 22 83–94. 10.1016/j.neuroimage.2003.10.051 15109999

[B113] Mulert C. Leicht G. Hepp P. Kirsch V. Karch S. Pogarell O. (2010). Single-trial coupling of the gamma-band response and the corresponding BOLD signal. *Neuroimage* 49 2238–2247. 10.1016/j.neuroimage.2009.10.058 19878729

[B114] Musso F. Brinkmeyer J. Mobascher A. Warbrick T. Winterer G. (2010). Spontaneous brain activity and EEG microstates. A novel EEG/fMRI analysis approach to explore resting-state networks. *Neuroimage* 52 1149–1161. 10.1016/j.neuroimage.2010.01.093 20139014

[B115] Mwansisya T. E. Hu A. Li Y. Chen X. Wu G. Huang X. (2017). Task and resting-state fMRI studies in first-episode schizophrenia: a systematic review. *Schizophr. Res.* 189 9–18. 10.1016/J.SCHRES.2017.02.026 28268041

[B116] Nagai Y. Critchley H. D. Featherstone E. Fenwick P. B. C. Trimble M. R. Dolan R. J. (2004). Brain activity relating to the contingent negative variation: an fMRI investigation. *Neuroimage* 21 1232–1241. 10.1016/j.neuroimage.2003.10.036 15050551

[B117] Neuner I. Rajkumar R. Brambilla C. R. Ramkiran S. Ruch A. Orth L. (2019). Simultaneous PET-MR-EEG: technology, challenges and application in clinical neuroscience. *IEEE Trans. Radiat Plasma Med. Sci.* 3 377–385. 10.1109/TRPMS.2018.2886525

[B118] Nikravan M. Ebrahimzadeh E. (2021). “Time-frequency analysis in EEG for the treatment of major depressive disorder using rTMS,” in *Proceedings of the 2021 Asia-Pacific International Symposium on Electromagnetic Compatibility (APEMC)*, Bali. 10.1109/APEMC49932.2021.9597080

[B119] Nikravan M. Ebrahimzadeh E. Izadi M. R. Mikaeili M. (2016). Toward a computer aided diagnosis system for lumbar disc herniation disease based on MR images analysis. *Biomed. Eng. Appl. Basis Commun.* 28:1650042. 10.4015/S1016237216500423

[B120] Nöth U. Laufs H. Stoermer R. Deichmann R. (2012). Simultaneous electroencephalography-functional MRI at 3 T: an analysis of safety risks imposed by performing anatomical reference scans with the EEG equipment in place. *J Magn. Reson. Imaging* 35 561–571. 10.1002/JMRI.22843 22002900

[B121] Olejniczak P. (2006). Neurophysiologic basis of EEG. *J. Clin. Neurophysiol.* 23 186–189. 10.1097/01.WNP.0000220079.61973.6C 16751718

[B122] Omidvarnia A. Kowalczyk M. A. Pedersen M. Jackson G. D. (2019). Towards fast and reliable simultaneous EEG-fMRI analysis of epilepsy with automatic spike detection. *Clin. Neurophysiol.* 130 368–378. 10.1016/j.clinph.2018.11.024 30669013

[B123] Opitz B. Mecklinger A. von Cramon D. Y. Kruggel F. (1999). Combining electrophysiological and hemodynamic measures of the auditory oddball. *Psychophysiology* 36 142–147. 10.1017/s0048577299980848 10098390

[B124] Pareto D. Sastre-Garriga J. Alonso J. Galán I. Arévalo M. J. Renom M. (2018). Classic block design “pseudo”-resting-state fMRI changes after a neurorehabilitation program in patients with multiple sclerosis. *J. Neuroimaging* 28 313–319. 10.1111/JON.12500 29400912

[B125] Patel M. J. Andreescu C. Price J. C. Edelman K. L. Reynolds I. I. I. C. F. Aizenstein H. J. (2015). Machine learning approaches for integrating clinical and imaging features in late-life depression classification and response prediction. *Int. J. Geriatr. Psychiatry* 30 1056–1067. 25689482 10.1002/gps.4262 PMC4683603

[B126] Peng H. Xia C. Wang Z. Zhu J. Zhang X. Sun S. (2019). Multivariate pattern analysis of EEG-based functional connectivity: a study on the identification of depression. *IEEE Access* 7 92630–92641. 10.1109/ACCESS.2019.2927121

[B127] Pisauro M. A. Fouragnan E. Retzler C. Philiastides M. G. (2017). Neural correlates of evidence accumulation during value-based decisions revealed via simultaneous EEG-fMRI. *Nat. Commun.* 8:15808. 10.1038/ncomms15808 28598432 PMC5472767

[B128] Pittau F. Dubeau F. Gotman J. (2012). Contribution of EEG/fMRI to the definition of the epileptic focus. *Neurology* 78 1479–1487. 10.1212/WNL.0b013e3182553bf7 22539574 PMC3345614

[B129] Raichle M. E. Snyder A. Z. (2007). A default mode of brain function: a brief history of an evolving idea. *Neuroimage* 37 1083–1090. 10.1016/j.neuroimage.2007.02.041 17719799

[B130] Rajkumar R. Kops E. R. Mauler J. Tellmann L. Lerche C. Herzog H. (2017). Simultaneous trimodal PET-MR-EEG imaging: do EEG caps generate artefacts in PET images? *PLoS One* 12:e0184743. 10.1371/JOURNAL.PONE.0184743 28902890 PMC5597218

[B131] Rosenkranz K. Lemieux L. (2010). Present and future of simultaneous EEG-fMRI. *Magn. Reson. Mater. Phys. Biol. Med.* 23 309–316. 10.1007/s10334-009-0196-9 20101434

[B132] Russo V. Paciocco A. Affinito A. Roscigno G. Fiore D. Palma F. (2018). Aptamer-miR-34c conjugate affects cell proliferation of non-small-cell lung cancer cells. *Mol. Ther. Nucleic Acids* 13 334–346. 10.1016/J.OMTN.2018.09.016 30340138 PMC6197774

[B133] Sadjadi S. M. Ebrahimzadeh E. Soltanian-Zadeh H. (2022). “fMRI functional connectivity analysis for localizing epileptic focus,” in *Proceedings of the 30th International Conference on Electrical Engineering*, (La Vergne, TN: ICEE).

[B134] Sadjadi S. M. Ebrahimzadeh E. Shams M. Seraji M. Soltanian-Zadeh H. (2021). Localization of epileptic foci based on simultaneous EEG–fMRI data. *Front. Neurol.* 12:645594. 10.3389/fneur.2021.645594 33986718 PMC8110922

[B135] Salek-Haddadi A. Friston K. J. Lemieux L. Fish D. R. (2003). Studying spontaneous EEG activity with fMRI. *Brain Res. Rev.* 43 110–133. 14499465 10.1016/s0165-0173(03)00193-0

[B136] Salmon E. Ir C. B. Hustinx R. (2015). Pitfalls and limitations of PET/CT in brain imaging. *Semin. Nucl. Med.* 45 541–551. 10.1053/J.SEMNUCLMED.2015.03.008 26522395

[B137] Scheeringa R. Petersson K. M. Kleinschmidt A. Jensen O. Bastiaansen M. C. M. (2012). EEG alpha power modulation of fMRI resting-state connectivity. *Brain Connect.* 2 254–264. 22938826 10.1089/brain.2012.0088 PMC3621304

[B138] Scrivener C. L. (2021). When is simultaneous recording necessary? A guide for researchers considering combined eeg-fmri. *Front. Neurosci.* 15:774. 10.3389/fnins.2021.636424 34267620 PMC8276697

[B139] Scrivener C. L. Reader A. T. (2022). Variability of EEG electrode positions and their underlying brain regions: visualizing gel artifacts from a simultaneous EEG-fMRI dataset. *Brain Behav.* 12:e2476. 10.1002/brb3.2476 35040596 PMC8865144

[B140] Seraji M. Mohebbi M. Safari A. Krekelberg B. (2021). Multiple sclerosis reduces synchrony of the magnocellular pathway. *PLoS One* 16:e0255324. 10.1371/journal.pone.0255324 34437558 PMC8389379

[B141] Shah N. J. Oros-Peusquens A. M. Arrubla J. Zhang K. Warbrick T. Mauler J. (2013). Advances in multimodal neuroimaging: hybrid MR-PET and MR-PET-EEG at 3 T and 9.4 T. *J. Magn. Reson.* 229 101–115. 10.1016/J.JMR.2012.11.027 23317760

[B142] Snyder A. Z. Abdullaev Y. G. Posner M. I. Raichle M. E. (1995). Scalp electrical potentials reflect regional cerebral blood flow responses during processing of written words. *Proc. Natl. Acad. Sci. U.S.A.* 92 1689–1693. 10.1073/pnas.92.5.1689 7878041 PMC42585

[B143] Soddu A. Vanhaudenhuyse A. Demertzi A. Bruno M. A. Tshibanda L. Di H. (2011). Resting state activity in patients with disorders of consciousness. *Funct. Neurol.* 26 37–43. 21693087 PMC3814510

[B144] Spencer K. M. Nestor P. G. Niznikiewicz M. A. Salisbury D. F. Shenton M. E. McCarley R. W. (2003). Abnormal neural synchrony in schizophrenia. *J. Neurosci.* 23 7407–7411. 12917376 10.1523/JNEUROSCI.23-19-07407.2003 PMC2848257

[B145] Srivastava G. Crottaz-Herbette S. Lau K. M. Glover G. H. Menon V. (2005). ICA-based procedures for removing ballistocardiogram artifacts from EEG data acquired in the MRI scanner. *Neuroimage* 24 50–60. 10.1016/J.NEUROIMAGE.2004.09.041 15588596

[B146] Steyrl D. Müller-Putz G. R. (2019). Artifacts in EEG of simultaneous EEG-fMRI: pulse artifact remainders in the gradient artifact template are a source of artifact residuals after average artifact subtraction. *J. Neural Eng.* 16:016011. 10.1088/1741-2552/AAEC42 30523809

[B147] Sur S. Sinha V. V. K. (2009). Event-related potential: an overview. *Ind. Psychiatry J.* 18:70. 10.4103/0972-6748.57865 21234168 PMC3016705

[B148] Tehrani N. Wilson W. Pittman D. J. Mosher V. Peedicail J. S. Aghakhani Y. (2021). Localization of interictal discharge origin: a simultaneous intracranial electroencephalographic–functional magnetic resonance imaging study. *Epilepsia* 62 1105–1118. 10.1111/epi.16887 33782964

[B149] Thees S. Blankenburg F. Taskin B. Curio G. Villringer A. (2003). Dipole source localization and fMRI of simultaneously recorded data applied to somatosensory categorization. *Neuroimage* 18 707–719. 10.1016/s1053-8119(02)00054-x 12667848

[B150] Tsuchimoto S. Shibusawa S. Mizuguchi N. Kato K. Ebata H. Liu M. (2017). Resting-state fluctuations of EEG sensorimotor rhythm reflect BOLD activities in the pericentral areas: a simultaneous EEG-fMRI study. *Front. Hum. Neurosci.* 11:356. 10.3389/FNHUM.2017.00356 28729830 PMC5498521

[B151] Waites A. B. Shaw M. E. Briellmann R. S. Labate A. Abbott D. F. Jackson G. D. (2005). How reliable are fMRI–EEG studies of epilepsy? A nonparametric approach to analysis validation and optimization. *Neuroimage* 24 192–199. 10.1016/j.neuroimage.2004.09.005 15588610

[B152] Wang L. Saalmann Y. B. Pinsk M. A. Arcaro M. J. Kastner S. (2012). Electrophysiological low-frequency coherence and cross-frequency coupling contribute to BOLD connectivity. *Neuron* 76 1010–1020. 10.1016/j.neuron.2012.09.033 23217748 PMC3531830

[B153] Yin S. Liu Y. Ding M. (2016). Amplitude of sensorimotor mu rhythm is correlated with BOLD from multiple brain regions: a simultaneous EEG-fMRI study. *Front. Hum. Neurosci.* 10:364. 10.3389/FNHUM.2016.00364 27499736 PMC4957514

[B154] Yu Q. Wu L. Bridwell D. A. Erhardt E. B. Du Y. He H. (2016). Building an EEG-fMRI multi-modal brain graph: a concurrent EEG-fMRI study. *Front. Hum. Neurosci.* 10:476. 10.3389/fnhum.2016.00476 27733821 PMC5039193

[B155] Yuan H. Phillips R. Wong C. K. Zotev V. Misaki M. Wurfel B. (2018). Tracking resting state connectivity dynamics in veterans with PTSD. *Neuroimage Clin.* 19 260–270. 10.1016/J.NICL.2018.04.014 30035020 PMC6051475

[B156] Zich C. Debener S. Kranczioch C. Bleichner M. G. Gutberlet I. De Vos M. (2015). Real-time EEG feedback during simultaneous EEG-fMRI identifies the cortical signature of motor imagery. *Neuroimage* 114 438–447. 25887263 10.1016/j.neuroimage.2015.04.020

[B157] Zotev V. Misaki M. Phillips R. Wong C. K. Bodurka J. (2018a). Real-time fMRI neurofeedback of the mediodorsal and anterior thalamus enhances correlation between thalamic BOLD activity and alpha EEG rhythm. *Hum. Brain Mapp.* 39 1024–1042. 10.1002/HBM.23902 29181883 PMC6866453

[B158] Zotev V. Phillips R. Misaki M. Wong C. K. Wurfel B. E. Krueger F. (2018b). Real-time fMRI neurofeedback training of the amygdala activity with simultaneous EEG in veterans with combat-related PTSD. *Neuroimage Clin.* 19 106–121. 10.1016/j.nicl.2018.04.010 30035008 PMC6051473

[B159] Zotev V. Phillips R. Yuan H. Misaki M. Bodurka J. (2014). Self-regulation of human brain activity using simultaneous real-time fMRI and EEG neurofeedback. *Neuroimage* 85 985–995. 10.1016/J.NEUROIMAGE.2013.04.126 23668969

